# The role of streptavidin and its variants in catalysis by biotinylated secondary amines†

**DOI:** 10.1039/d1ob01947c

**Published:** 2021-11-15

**Authors:** Alexander R. Nödling, Nicolò Santi, Raquel Castillo, Magdalena Lipka-Lloyd, Yi Jin, Louis C. Morrill, Katarzyna Świderek, Vicent Moliner, Louis Y. P. Luk

**Affiliations:** School of Chemistry, Main Building, Cardiff University Cardiff CF10 3AT UK LukLY@cardiff.ac.uk; Department de Química Física i Analítica, Universitat Jaume I Castellón 12071 Spain swiderek@uji.es moliner@uji.es; Cardiff Catalysis Institute, School of Chemistry, Main Building, Cardiff University Cardiff CF10 3AT UK

## Abstract

Here, we combine the use of host screening, protein crystallography and QM/MM molecular dynamics simulations to investigate how the protein structure affects iminium catalysis by biotinylated secondary amines in a model 1,4 conjugate addition reaction. Monomeric streptavidin (M-Sav) lacks a quaternary structure and the solvent-exposed reaction site resulted in poor product conversion in the model reaction with low enantio- and regioselectivities. These parameters were much improved when the tetrameric host T-Sav was used; indeed, residues at the symmetrical subunit interface were proven to be critical for catalysis through a mutagenesis study. The use of QM/MM simulations and the asymmetric dimeric variant D-Sav revealed that both Lys121 residues which are located in the hosting and neighboring subunits play a critical role in controlling the stereoselectivity and reactivity. Lastly, the D-Sav template, though providing a lower conversion than that of the symmetric tetrameric counterpart, is likely a better starting point for future protein engineering because each surrounding residue within the asymmetric scaffold can be refined for secondary amine catalysis.

## Abstract 2

Here, we combine the use of host screening, protein crystallography and QM/MM molecular dynamics simulations to investigate how protein enviroment affects iminium catalysis by biotinylated secondary amines in a model 1,4 conjugate addition reaction.

## Graphical abstract

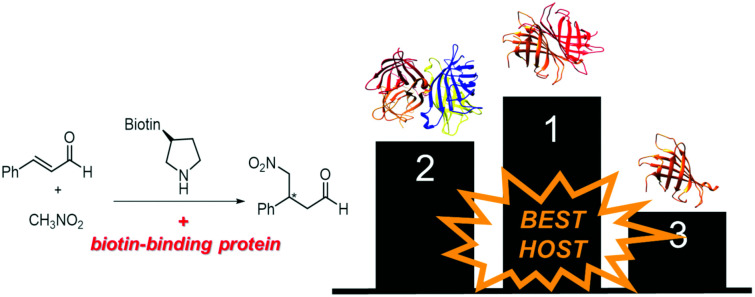

## Introduction

Organocatalysts are powerful auxiliaries used in the design of novel artificial enzymes.^1–3^ Various engineering approaches have been applied to facilitate organocatalysis within a protein scaffold including side-chain labeling,^4–8^ genetic code expansion,^2,9–14^ use of N-terminal prolines,^3,15,16^ computational design^17–20^ and the protein–ligand approach.^1,21–24^ To screen a large number of catalysts within a short timeframe, the protein–ligand approach is most frequently used.^1,21–24^ In this approach, ligands are covalently linked with chemical catalysts and then introduced to their binding proteins for activity test.^25–31^ One important consideration is to choose an appropriate protein partner because there are multiple homologues that bind the same ligand with high affinity, but their effect on chemical catalysis can be vastly different.^32–38^ Accordingly, detailed mechanistic studies must be conducted during the design of artificial enzymes.

**Fig. 1:**
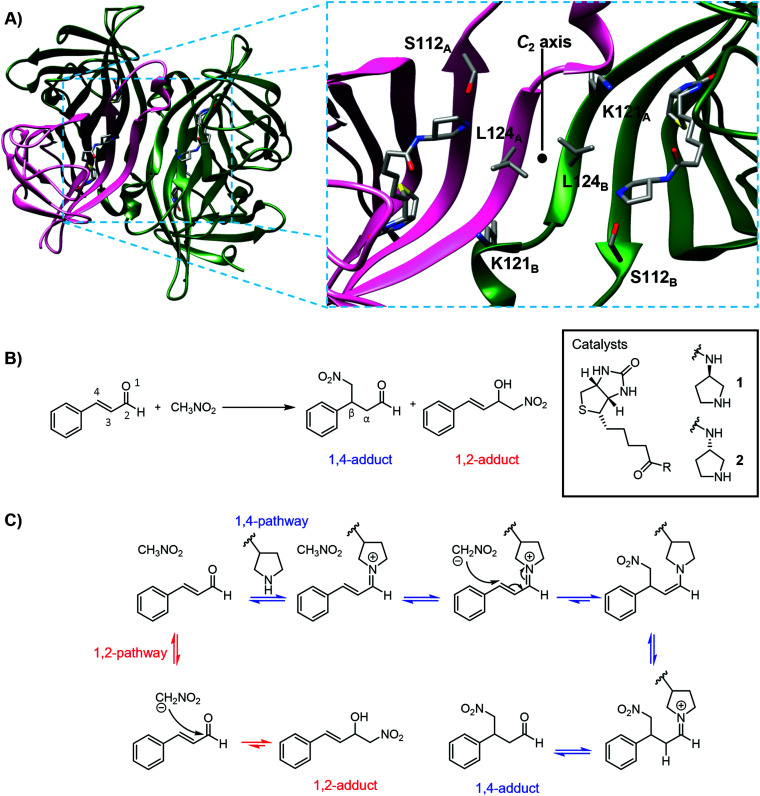
(A) Left: Cartoon of tetrameric streptavidin (T-Sav) containing ligand 1 (PDB: 6GH7); the monomer unit is shown in pink. Right: Close up of the *C*_2_-symmetric interunit interface with Ser112, Lys121, and Leu124 highlighted; two monomer units in the background are omitted for clarity. (B) Model reaction catalyzed by biotinylated catalysts 1 and 2 within the tetrameric streptavidin (T-Sav), showing the desired 1,4-addition product and 1,2-addition side-product. (C) Mechanistic scheme leading to the desired 1,4-addition and 1,2-addition side-product.

The importance of choosing an appropriate host has been demonstrated in the use of biotin-binding proteins for artificial enzyme design. Tetrameric streptavidin (T-Sav) has been popularly used, engineered and evolved for hosting reactions that are mediated by biotin-appended chemical catalysts.^25–29,32,33,39–43^ In each T-Sav, there are four biotin-binding sites whose arrangement locates the catalyzed reactions at the interface of two subunits which has a *C*_2_ symmetry (Fig. 1A).^21,23,36,40,42,44–47^ Consequently, changing both the surrounding residues and the degree of binding site occupancy can affect the reaction reactivity and stereoselectivity.^36,48,49^ Furthermore, a switch in the biotin-binding scaffold has also shown to drastically affect the performance of the catalysis.^36,37^ In one scenario, the Ir-catalyzed hydrogen transfer reaction improved significantly when the monomeric subunits of T-Sav were dimerized and transformed into an asymmetrical scaffold (D-Sav) that hosts chemical catalysis without interference caused by (non-)cooperative binding.^36^ In the other scenario, the Rh(iii)-catalyzed lactam formation is enhanced only when T-Sav is replaced by an engineered monomeric counterpart M-Sav; this phenomenon was attributed to enhanced solvent exposure of the bulky catalyst (Fig. 1A).^37^ These studies indicated that a change of the protein host could greatly affect the outcome of the chemical transformations mediated by metal catalysts, but equivalent studies on protein-hosted organocatalysis have not been conducted.

We have recently reported an organocatalytic 1,4 conjugate addition of nitromethane to cinnamaldehydes within a protein environment through adaptation of the streptavidin–biotin technology (Fig. 1B & C).^21^ In this system, biotin was covalently modified with pyrrolidine groups, resulting in ligands 1 and 2 which differ by one stereocenter (Fig. 1B). The protein T-Sav could not mediate the model reaction and catalysis by the ligands themselves was not stereoselective (Table 1, entries 10 and 11), yet the T-Sav : 1 complex favors the formation of the *S* enantiomer (91 : 9), whereas the T-Sav : 2 complex favors the formation of the *R* counterpart (24 : 76).^21^ Moreover, this protein-hosted system accepts various types of substrates for iminium^21,50^ or enamine catalysis.^51^ Whilst promising, the role of the T-Sav residues in proximity to the pyrrolidine has not been investigated. Importantly, whether T-Sav is the most suitable host for iminium organocatalysis has not been verified, yet such information is fundamental to the creation of a high-performance artificial enzyme.

**Table 1:** Organocatalytic reaction (1) hosted by variants of streptavidin (Sav)

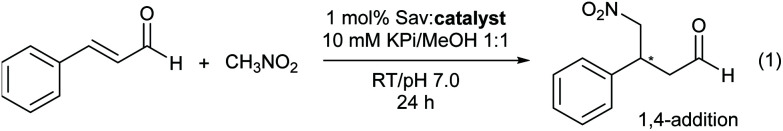					
Entry	Catalyst	Host	Conversion^a^/%	Ratio 1,4- : 1,2-addition	er (*S* : *R*)
1	1	M-Sav (WT)^b^	22	1.4 : 1.0	N.D.
2	2	M-Sav (WT)^b^	27	2.9 : 1.0	
3	2	M-Sav (Y111S)^b^	36	1.3 : 1.0	
4	2	M-Sav (Y111T)^b^	33	1.5 : 1.0	
5	2	M-Sav (Y111V)^b^	28	0.6 : 1.0	
6	2	M-Sav (Y111K)^b^	46	2.1 : 1.0	
7	2	M-Sav (Y111A)^b^	33	1.5 : 1.0	
8	2	M-Sav (E120L)^b^	61	9.2 : 1.0	40 : 60
9	2	M-Sav (Y111S E120L)^b^	41	1.2 : 1.0	N.D.
10	1	—	57	8.5 : 1.0	48 : 52
11	—	T-rSav (WT)^f^	21	1.2 : 1.0	N.D.
12	1^c^	T-Sav (WT)^d^	30	>5.0 : 1.0	80 : 20
13	1	T-Sav (WT)	80	>15.0 : 1.0	91 : 9
14	1	T-rSav (WT)^e^	87	6.3 : 1.0	90 : 10
15	1	T-rSav (WT)^f^	71	6.9 : 1.0	80 : 20
16	2^c^	T-Sav (WT)	15	>2.0 : 1.0	24 : 76
17	2	T-Sav (WT)	71	>13.2 : 1.0	33 : 67
18	1	T-rSav S112V^f^	88	3.9 : 1.0	63 : 37
19	1	T-rSav S112E^f^	43	3.3 : 1.0	76 : 24
20	1	T-rSav S112Y^f^	77	4.9 : 1.0	53 : 47
21	1	T-rSav L124E^f^	23	0.8 : 1.0	51 : 49
22	1	T-rSav L124K^f^	80	2.2 : 1.0	53 : 47
23	1	T-rSav L124W^f^	92	>17.4 : 1.0	89 : 11
24	1	T-rSav K121A^f^	58	8.7 : 1.0	66 : 34
25	1	T-rSav K121M^f^	98	11.3 : 1.0	80 : 20
26	1	T-rSav K121R^f^	77	6.0 : 1.0	75 : 25
27	1	D-Sav SARK^e^	38	3.8 : 1.0	75 : 25
28	1	D-Sav SKRA^e^	<5	1.4 : 1.0	N.D.

a Conversion refers to the overall conversion of cinnamaldehyde to the 1,4- and 1,2-addition products that was performed in triplicate (see Fig. S1 for exemplified conversion evaluation, Tables S1–3 for site-directed mutagenesis primer sequences, experimental details on Pg S17–20, Pg S36–38 & Pg S52, and chiral LC results on Pg S43–51†).

b Additional protein purification using a Ni-affinity column (see Pg S14–16†).

c The reaction was performed without an organic solvent (MeOH) for 42 h. Its addition most likely helped in preventing cinnamaldehyde from precipitation and affecting the hydration ratio, thus changing the substrate accessibility for reactions and the 1,4- : 1,2-adduct ratio.^21^

d Conversion of the reaction catalyzed by T-Sav : biotin was found to be <3%.^21^

e Additional protein purification using an iminobiotin column (details on T-Sav and D-Sav purification on Pg S8–13†).

f Only purified by washing, denaturing and dialysis of the insoluble cell pellet. In line with the reports on different purity grade artificial metalloenzymes;^47,53^ slightly lower yields and enantioselectivities compared to the purified counterparts are observed (see also Pg S8–11†). T-Sav = tetrameric core streptavidin (see Pg S5† for a detailed sequence). T-rSav = tetrameric streptavidin with a “reduced” sequence.^35^ M-Sav = monomeric streptavidin.^34^ D-Sav = dimeric streptavidin.^36^ N.D. = not determined.

Here, we performed mechanistic investigations on how a protein host affects iminium catalysis. We began by testing the monomeric M-Sav as the host; the reaction lacks both enantio- and regioselectivity despite numerous optimization attempts, and protein X-ray crystallography analysis revealed that the catalyst is solvent exposed. Site-directed mutagenesis, Quantum Mechanics/Molecular Mechanics (QM/MM) analysis of T-Sav and the use of the asymmetric host D-Sav have indicated that intersubunit residues including Lys121 control both the reactivity and stereoselectivity. Even though the current D-Sav template provides a lower conversion than that of the symmetrical counterpart, we envisioned that an asymmetric scaffold would facilitate fine-tuning and thus it is likely a better starting point for future protein engineering work.

## Results and discussion

### Exploration of monomeric Sav hosts

The monomeric variant M-Sav was tested as an alternative host for iminium catalysis. Although it lacks the subunit interface and shares moderate sequence identity with T-Sav (55%), M-Sav has high affinity to biotin (*K*_D_ = 2.8 nM).^34^ M-Sav was shown to be minimally active for iminium catalysis,^50^ and its use as a host also resulted in poor chemical transformation in the model organocatalytic reaction (Fig. 1B and Table 1). Both the conversion and regioselectivity dropped when ligand 1 was introduced to M-Sav; cinnamaldehyde was converted to only a small amount of the product (13% as the 1,4-addition product) with nearly the same amount of the 1,2-addition side-product formed (9%, entries 1 & 10–16; also see Fig. 1B & S1†). Catalysis by 2 in M-Sav was mildly improved (20% 1,4-addition product, entry 2) but there remained a substantial amount of the 1,2-addition side product (7%). The melting temperature of M-Sav increased significantly when ligand 1 or 2 was included (+20 °C) as assessed by circular dichroism spectroscopy (Fig. S2†), implying its binding to the biotinylated catalysts.^37,51^ Although the reaction conditions have been heavily screened (buffer composition and pH, Tables S8–S10†), conversions remained low and regioselectivity (1,4- *vs.* 1,2-addition) was lacking.

**Fig. 2:**
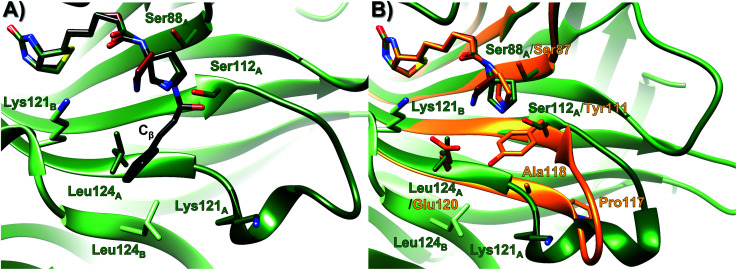
Overlay of the organocatalytic system T-Sav : 1 (green, PDB 6GH7)^22^ with (A) ligand 2 (red, PDB 7NLV, 1.29 Å) and the tetrahedral intermediate (black, QM/MM simulation), and (B) ligand 2 (red) and M Sav : 2 (orange, PDB 6ZYT, 1.80 Å). See Tables S4–6† for crystallization conditions and details.

To understand how a switch in the protein host causes a drastic change in catalysis, the X-ray crystallography structures of M-Sav : 2 and T-Sav : 2 were obtained at a resolution of 1.80 and 1.29 Å respectively (Fig. 2 and Tables S4–S6†), and subsequently site-directed mutagenesis analysis was conducted. Tyr111 in M-Sav : 2 is a relatively bulky residue located in proximity to the secondary amine motif and corresponds to Ser112 in T-Sav : 2. Replacing Tyr111 with other residues including serine, threonine, valine, lysine and alanine did not improve the conversion or the regioselectivity (entries 3–7). Glu120 in M-Sav : 2 is in proximity and spatially corresponds to Leu124 in T-Sav : 2. Though modifying Glu120 slightly improved the overall yield of the 1,4-addition product, the enantioselectivity of the catalysis remained low (entry 8). These observations are in contrast to a recent study which showed that M-Sav is a better host for Rh-catalyzed lactam synthesis.^37^ One noticeable difference is that the rhodium-based catalyst is bulky and has been observed to prefer an exposed active site for reaction, whereas the organocatalytic iminium intermediate typically requires a hydrophobic environment to drive and steer the selectivity of the catalytic cycle.^52^

To understand why T-Sav is a superior host for the model organocatalytic reaction,^21^ the ratio of biotinylated ligand 1 to their binding site was investigated. The reaction yield was found to be 46% when an average of one catalyst occupied one of the tetrameric T-Sav hosts (entry 1, Table 2). When two of the binding sites were occupied, the conversion only increased up to 61%, instead of doubling to 92% (entry 2). Eventually, the yield increased to 81% when four of the binding sites were occupied by the catalyst (entries 3 and 4). The non-linear increase in yield implied that each T-Sav binding site cannot mediate iminium catalysis simultaneously and the intersubunit interface can only host one chemical transformation event at a time.

**Table 2:** Conversion % and er for the 1,4-additions at different T-Sav : 1 ratios

Entry	Equivalent 1	Conversion (%)	er (*S* : *R*)
1	1	46	87 : 13
2	2	61	82 : 17
3	3	65	87 : 13
4	4	81	87 : 13

For further investigations, site-directed mutagenesis was conducted by the use of a truncated variant termed “reduced streptavidin” (T-rSav, see Pg S8–11† for preparation).^35^ In previous and current crystallographic studies, Ser112 was found to be in a hydrogen-bonding distance with the reacting nitrogen atom of the pyrrolidine motif (Fig. 2A).^21^ When this residue was replaced by valine, glutamate or tyrosine, there was either a drop in conversion (71% to 43%, Table 1, entries 15 *vs.* 19) or enantioselectivity (80 : 20 to 53 : 47 *S* : *R*, Table 1, entries 15 *vs.* 20). Notably, the substrate was also converted into the 1,2-addition byproduct by these Ser112 variants, being reminiscent of the reactions hosted by M-Sav and its variants. Altogether, this suggests that Ser112 helps in positioning the intermediate for nucleophilic addition at the C_β_ position, improving both the regio- and enantioselectivities of the reaction.

Previous MD analysis indicated that Leu124 and Lys121 are located close to the tetrahedral intermediate.^21,28,29^ When Leu124 was changed to charged residues such as lysine and glutamate, the enantioselectivity vanished and a notable amount of the 1,2-addition side-product was observed (entries 21 and 22 in Table 1). In contrast, the conversion and stereoselectivity were mildly improved when the T-rSav-L124W variant was used as a host, indicating the importance of placing a bulky and hydrophobic residue at this position (entry 23). The Lys121 residues in both T-Sav subunits (namely SavA and SavB, see below) are also in close proximity to the reacting centers.^21,28,29^ To test their role(s), Lys121 was converted to alanine, methionine and arginine for analysis. In the reaction hosted by T-rSav-K121A, both the yield and enantioselectivity were vastly reduced (entry 24). In contrast, substituting Lys121 with methionine or arginine does not affect the performance of the reaction, implying that the size of the sidechain, rather than its electrostatic properties, is responsible for the observed selectivity (entries 25 and 26). Indeed, T-rSav-K121M provided a higher product conversion, yet the enantioselectivity remained similar to that of the wild-type host. Notably, the Lys121 variants resulted in low 1,2-byproduct formation, indicating that this residue does not improve the regioselectivity of the reaction but plays a role in guiding how the nucleophile (deprotonated nitromethane) approaches the C-4 of the iminium intermediate (Fig. 1B).

### Use of dimeric Sav as the host

**Fig. 3:**
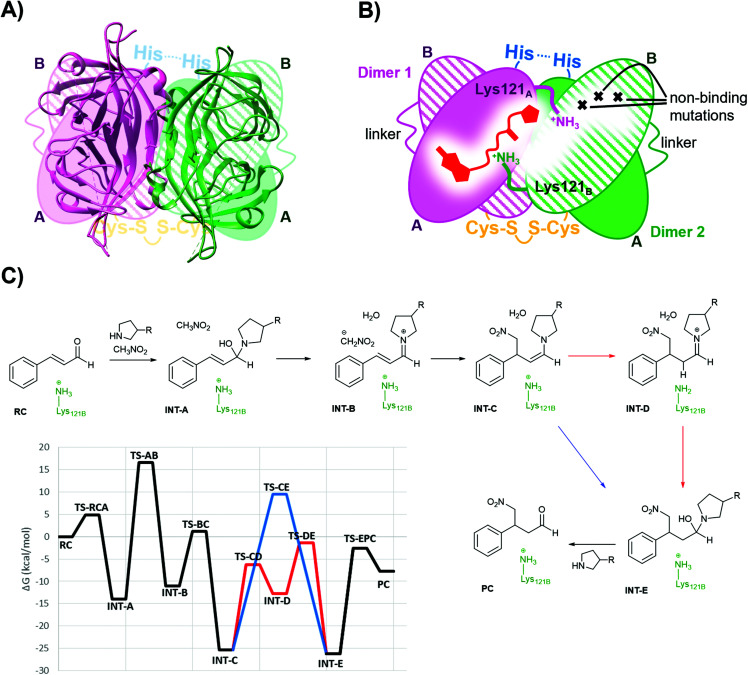
(A) Overlay of the D-Sav cartoon structure (PDB: 6S50) and a simplified schematic representation. (B) Schematic representation of one intersubunit interface in D-Sav between dimer A and dimer B including the monovalent binding of 1. (C) Schematic of the crucial steps in the reaction hosted by the T-Sav derived from the QM/MM free energy surfaces, and the M06-2X:AM1/MM free energy profile derived from the exploration of the free energy surfaces. Steps C to E are assisted by Lys121B (in red) or by a water molecule (in blue).

Single chain dimeric streptavidin (D-Sav) is a new family of engineered variants that enables independent mutagenesis of the two neighbouring streptavidin subunits, and it is used here to investigate the role of the intersubunit residues Lys121s in catalyzing the secondary amine organocatalytic reaction. According to a recent report by Wu *et al.*,^36^ D-Sav was achieved by connecting two subunits of streptavidin through a peptide linker (26-residue), resulting in a single chain monomer that carries two equivalents of biotin-binding modules, SavA and SavB (Fig. 3A and B). An intermolecular disulfide bond formed through an H127C mutation at SavB covalently connects two of the single chain monomers together, yielding the dimeric protein variant. D-Sav enables asymmetric genetic tuning of the biotin-binding vestibule (Fig. 3B), because SavB possesses additional mutations (N23A/S27D/D128A), prohibiting key H-bonding interactions needed for ligand binding. Consequently, each monomer is monovalent with the ligand binding only to the SavA module and avoids (non-)cooperative multiple site occupancy.

For this work, two asymmetric D-Sav variants were tested: SARK and SKRA, where the first two letters code for residues 112 and 121 in SavA and the last two for 112 and 121 in SavB (Fig. 2). Only D-Sav-SARK provides a measurable conversion with significant enantioselectivity, whereas D-Sav-SKRA could not provide a detectable yield (entries 27 and 28). Accordingly, this suggests that Lys121 in the neighbouring unit (*i.e.* Lys121B in SavB) plays a role in improving the reactivity and stereoselectivity; indeed, the QM/MM MD simulations carried out in the present study also suggest that Lys121B in the neighbouring subunit plays a role in guiding how the nucleophile (deprotonated nitromethane) approaches the iminium intermediate (see below). Nevertheless, we noticed that the conversion hosted by D-Sav-SARK is noticeably lower than that by the wild type T-Sav with substantial conversion of the 1,2-byproduct (entry 27). Overlaying the crystal structures of D-Sav and T-Sav indicated that the relative distance between the two binding sites differed moderately (Fig. S3†). This structural change is likely a consequence of protein engineering and may contribute to the decrease in reaction yield, and thus further investigations *via* an alternative QM/MM approach were conducted (see below).

### Molecular dynamics simulations

Derived from the free energy surfaces explored for every single chemical step at the M06-2X:AM1/MM level (Fig. 3C, S4 & S5 and Page S26–34†), the reaction mechanism and the corresponding free energy profile are outlined in Fig. 3C (see the ESI†). Our computational studies have generated a model where the geometry of the tetrahedral intermediate INT-A overlays well with the X-ray crystallographic structures. The reaction can take place in five to six steps depending on whether the protein host is included in the chemical transformation. In agreement with the previous work,^54^ the transformation from INT-A to INT-B is rate-limiting requiring the nitromethane to be deprotonated and the iminium ion to be formed before the nucleophilic addition (INT-B to INT-C). Any attempt to look for alternative timing of the events did not render plausible results. The energy required for reaching TS-AB from RC was calculated to be 16.6 kcal mol^−1^, the highest among all steps while presenting an intrinsic activation free energy barrier of 30.5 kcal mol^−1^. Hence, this suggests that all the other chemical steps are not or only partially rate-limiting. Upon forming the enamine intermediate INT-C, the reaction can proceed through two paths: a concerted step assisted by a water molecule (and/or a phosphate group) or a step-wise process assisted by Lys121B located in the neighbouring subunit (*i.e.* SavB) (steps in blue and red in Fig. 3C, respectively). Of note, the Lys121B-assisted pathway resulted in lower activation energy barriers.

**Fig. 4:**
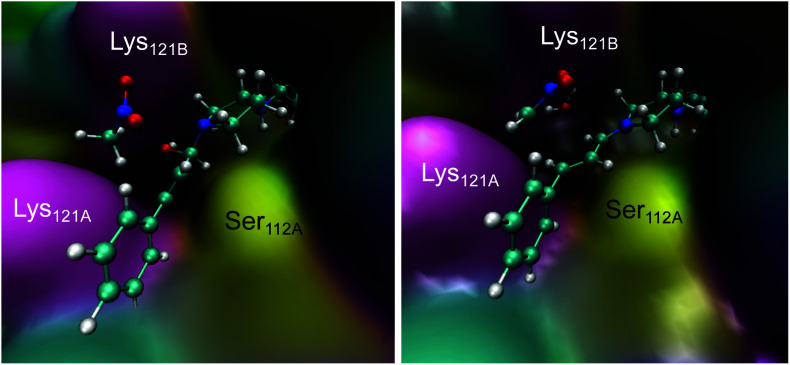
Representative snapshots of the INT-A (left) and INT-B (right) intermediates derived from the QM/MM MD simulations.

A dynamical model within a fully solvated protein was constructed to reveal the role of water molecules and residues at the subunit interface in catalysis (Fig. 4). The averaged structures of INT-A and INT-B obtained in the reaction path indicated that Ser112 and Lys121 assist in the positioning of the iminium intermediate for the nucleophilic addition at the Cβ position (Table S7 and Fig. 4, S6 & S7†). When Ser112 was converted into alanine, there was an enhancement of intermediate mobility. The carbonyl group became more solvent exposed, explaining the formation of the 1,2-addition byproduct in the experimental study (Table 1, entries 18–20). The Lys121Ala variant was found to provoke a significant increase in the mobility of INT-A, and hence the step of nucleophilic attack (INT-B to INT-C) can proceed from different orientations, causing a loss of enantioselectivity that is also observed experimentally (Table 1, entry 23, and Fig. S8†). In other words, the QM/MM study indicated that both Lys121A and Lys121B play a critical role in improving the reactivity and selectivity. While the QM/MM study confirmed the role of Lys121B in catalysis, it also contradicts the observation that there was a low but measurable activity when D-Sav-SARK serves as a host in which the corresponding Lys121A is replaced by an alanine residue. A possible explanation is that the role of Lys121A was replaced by neighboring residue(s) in this D-Sav variant which guides the nucleophile to interact with the intermediate in a stereoselective manner.

## Conclusions

Our mutational, crystallographic and mechanistic studies have indicated that the protein environment corresponding to the subunit interface of T-Sav is critical for catalysis by biotinylated secondary amines. This microenvironment controls the reaction conversion, regioselectivity (1,4- over 1,2-addition) and stereoselectivity (*S vs. R*). In contrast, the monomeric M-Sav contains a solvent-exposed site and was found to be a poor host for iminium catalysis. The use of asymmetric D-Sav and computational analysis have indicated that both Lys121A and Lys121B play a role in improving the stereoselectivity and reactivity. We believe that the D-Sav template is the most ideal starting point for future protein engineering work; although it gives a lower yield and selectivity in comparison to its tetrameric counterpart, its asymmetric feature will likely allow fine-tuning of the reaction scaffold, similar to many of the well-evolved artificial enzymes.^10,43,55–57^ Indeed, an initial enzyme often has poor activity but, upon optimization by direct evolution, both the yield and selectivity can be improved exponentially within a few rounds of iterations.^56^ By revealing the protein microenvironment relevant to secondary amine catalysis, the presented mechanistic study will find use in creating high-performance artificial enzymes.

## Author contributions

ARN, KS, VM, and LYPL designed the study. NS performed experimental work regarding M-Sav and the T-Sav : 1 ratio study. ARN performed all other experimental work regarding T-Sav, D-Sav and M-Sav E120L mutants. Crystallography was performed by ARN, NS, ML-L, and YJ. RC and KS performed MD simulations. ARN, KS, VM, and LYPL wrote the paper. All authors discussed and commented on the manuscript. LCM, KS, VM, and LYPL acquired funding.

## Conflicts of interest

There are no conflicts to declare.

## Supplementary Material

OB-019-D1OB01947C-s001
