# Supplementary material for: The role of streptavidin and its variants in catalysis by biotinylated secondary amines
Source: Org Biomol Chem. 2021 Nov 15;19(47):10424–31. doi: 10.1039/d1ob01947c (PMC8652411; doi:10.1039/d1ob01947c)
Supplement: OB-019-D1OB01947C-s001 [file OB-019-D1OB01947C-s001.pdf]

Electronic Supplementary Material (ESI) for Organic & Biomolecular Chemistry.
This journal is © The Royal Society of Chemistry 2021

Supporting Information

The Role of Streptavidin and its variants in Catalysis by Biotinylated Secondary Amines

Alexander R. Nödling,[a] Nicolò Santi,[a] Raquel Castillo,[b] Magdalena Lipka-Lloyd,[a] Yi Jin,[a] Louis C. Morrill,[b] Katarzyna Świderek,*[b] Vicent Moliner,*[b] Louis Y.P. Luk*[ac]

[a] School of Chemistry, Main Building, Cardiff University, Cardiff, CF10 3AT (UK)
[b] Department de Química Física i Analítica, Universitat Jaume I, Castellk, 12071 (Spain)
[c] Cardiff Catalysis Institute, School of Chemistry, Main Building, Cardiff University, Cardiff, CF10 3AT (UK)

*E-mail: lukly@cardiff.ac.uk
*E-Mail: swiderek@uji.es; moliner@uji.es

Table of Contents

1 General Information 5

2 Experimental Details for the Preparation and Purification of T-rSav and mutants. 8

2.1 Expression and Purification 8

2.2 T-rSav DNA sequence 9

2.3 Site-directed mutagenesis 9

3 Experimental Details for the Preparation and Purification of D-Sav and mutants. 12

3.1 Expression and Purification 12

3.2 D-Sav SARK DNA sequence 12

3.3 Site-directed mutagenesis 13

4 Experimental Details for the Preparation and Purification of M-Sav and mutants. 14

4.1 Expression and Purification 14

4.2 M-Sav DNA sequence 14

4.3 Site-directed mutagenesis 15

4.4 Circular Dichroism experiments 16

5 Experimental Details for the Activity and Selectivity Screening of Sav-based Hybrid Catalysts in the Michael Addition of Nitromethane to Cinnamaldehyde 17

5.1 ^{1}H NMR Based Screening for Yield – T-Sav 17

Runs in KP_i Buffer 17

Runs in KP_i Buffer / MeOH 17

Catalyst Background Runs 18

5.2 ^{1}H NMR Based Screening for Yield – T-rSav 18

General Procedure for all Runs 18

5.3 ^{1}H NMR Based Screening for Yield – D-Sav 18

General Procedure for all Runs 18

5.4 ^{1}H NMR Based Screening for Yield – M-Sav 19

General Procedure for all Runs 19

5.5 ^{1}H NMR Based Screening for Yield – Exemplary Analysis 19

5.6 Enantioselectivity Determination for all Sav Variants 20

6 Experimental Details for the Structure Determination of T-Sav:2 22

6.1 Procedure for Crystallization of T-Sav:2 22

6.2 X-ray data collection, processing and structure solution 22

6.3 Crystallographic Details for the Obtained Crystal Structure of T-Sav:2 23
7 Experimental Details for the Structure Determination of M-Sav:2 24
7.1 Procedure for Crystallization of M-Sav:2 24
7.2 X-ray data collection, processing and structure solution 24
7.3 Crystallographic Details for the Obtained Crystal Structure of M-Sav:2 25
8 Computational Methods 26
8.1 Computational Details of the Molecular Models Set Up 26
8.2 Computational details of the QM/MM simulations 27
8.3 Potential Energy Surfaces 28
8.4 Free Energy Surfaces 28
8.5 Spline Corrections 28
8.6 Computational Results 29
9 Plasmid maps 36
9.1 Plasmid map T-rSav (Addgene, Plasmid #17327) 36
9.1 Plasmid map M-Sav (Addgene, Plasmid #39860) 36
10 SDS-PAGE Gels of T-rSav and M-Sav 37
10.1 T-rSav SDS PAGE Gels 37
T-rSav Wildtype 37
T-rSav Exemplary Mutants 37
10.2 M-Sav SDS PAGE Gels 38
10.3 D-Sav SDS PAGE Gels 38
11 ESI-MS 39
11.1 ESI-MS of the Expressed T-rSav 39
11.2 ESI-MS of the Expressed M-Sav 40
11.3 ESI-MS of the Expressed D-Sav 40
12 CD spectra for M-Sav wt, M-Sav:1, M-Sav:2 and M-Sav:biotin 41
12.1 CD spectrum M-Sav wt 41
12.2 CD spectrum M-Sav:1 41
12.3 CD spectrum M-Sav:2 42
12.4 CD spectrum M-Sav:biotin 42
13 Chiral HPLC Data of Activity and Selectivity Screening 43
13.1 Cinnamaldehyde Runs using Different Streptavidin Constructs 43
Racemate S2 43
Product S2 with T-Sav:2 44

Product S2 with 1 44
Product S2 with 2 45
Product S2 with T-rSav:1 (wt) 45
Product S2 with T-rSav:1 (S112V) 46
Product S2 with T-rSav:1 (S112E) 46
Product S2 with T-rSav:1 (S112Y) 47
Product S2 with T-rSav:1 (K121A) 47
Product S2 with T-rSav:1 (K121M) 48
Product S2 with T-rSav:1 (K121R) 48
Product S2 with T-rSav:1 (L124E) 49
Product S2 with T-rSav:1 (L124K) 49
Product S2 with T-rSav:1 (L124W) 50
Product S2 with D-Sav:1 (SARK) 50
Product S2 with M-Sav:2 (E120L) 51
14 Additional Figures & Tables 52
14.1 Initial Screening Results M-Sav 52
15 References 54

1 General Information

Reactions were performed without precautions to exclude air. Reaction temperatures are stated as heating device temperature (e.g. oil bath, shaker, etc.), if not stated otherwise. Concentrations under reduced pressure were performed by rotary evaporation at 40°C at the appropriated pressure, unless otherwise noted. Deionized water was obtained by an *Elga PURELAB Option* system (15 MΩ·cm). Analytical and preparative Thin Layer Chromatography (TLC) was carried out with silica gel 60 F254 aluminium sheets from *Merck*. Detection was carried out using UV light (λ = 254 nm and 366 nm), followed by immersion in permanganate or cerium ammonium molybdate staining solution with subsequent development via careful heating with a heat gun. Flash column chromatography was performed using silica gel (pore size 60 Å, 0.040-0.063 mm).

Cinnamaldehyde for 1,4-addition reactions with **T-rSav** was used as received from commercial suppliers. For all other reactions it was purified by washes with sodium bicarbonate pH 8.3, subsequent drying with magnesium sulfate and stored under inert atmosphere at −23 °C. All other solvents and reagents were obtained from commercial sources and used as received.

Plasmid miniprep-kit and gel extraction-kit were purchased from *Qiagen*. DNA oligos were purchased from *Sigma-Aldrich*.

T-Sav (Streptavidin *Streptomyces avidinii* recombinant, tetramer, M_w ≈ 52 kDa, "core" Streptavidin with amino acids 13-139) was obtained commercially from *ProSpec* (PRO-791) as lyophilized powder in 10 mM KP_i pH 6.5 and stored at −23 °C upon receipt until further use. According to the supplier T-Sav has the following amino acid sequence:

```
MAEAGITGTWYNQLGSTFIVTAGADGALTGTYESAVGNAESRYVLT
GRYDSAPATDGSGTALGWTVAWKNNYRNAHSATTWSGQYVGGAEAR
INTQWLLTSGTTEANAWKSTLVGHDTFTKVKPSAAS
```

The plasmid for T-rSav (encoding for a Streptavidin *Streptomyces avidinii* recombinant, tetramer, M_w ≈ 50 kDa, "reduced" Streptavidin with amino acids 16-133) was obtained as gift from Takeshi Sano (pTSA-13, Addgene plasmid #17327, http://n2t.net/addgene:17327, RRID:Addgene_17327). The gene encoding for T-rSav translates to the following amino acid sequence:

```
MGITGTWYNQLGSTFIVTAGADGALTGTYESAVGNAESRYVLTGRY
DSAPATDGSGTALGWTVAWKNNYRNAHSATTWSGQYVGGAEARINT
QWLLTSGTTEANAWKSTLVGHDTFTKV*
```

The plasmid for M-Sav (encoding for a monomeric Streptavidin/Avidin recombinant, M_w ≈ 16 kDa) was obtained as gift from Sheldon Park (pRSET-mSA, Addgene plasmid #39860, http://n2t.net/addgene:39860, RRID:Addgene_39860). The gene encoding for M-Sav translates to the following amino acid sequence:

MGSSHHHHHHSQDLASAEAGITGTWYNQSGSTFTVTAGADGNLTGQ
YENRAQGTGCQNSPYTLTGRYNGTKLEWRVEWNNSTENCHSRTEWR
GQYQGGAEARINTQWNLTYEGGSGPATEQGQDTFTKVKPSAASGSD
YKDDDDK*

The plasmid for D-Sav (encoding for a mono-valent single-chain dimeric Streptavidin dimer forming recombinant with the SARK mutations, $M_w \approx 70$ kDa)[1] was obtained as gift from Thomas Ward. The gene encoding for D-Sav translates to the following amino acid sequence:

MASMTGGQQMGRDQAGITGTWYNQLGSTFIVTAGADGALTGTYESA
VGNAESRYVLTGRYDSAPATDGSGTALGWTVAWKNNYRNAHSATTW
SGQYVGGAEARINTQWLLTSGTTEANAWASTLVGHDTFTKVKPSAA
SIDAAKKAGVNNGNPLDAVQQGSGGGNGGGNGGGNGGGNIDGRGGG
NASMTGGQQMGRDQAGITGTWYAQLGDTFIVTAGADGALTGTYVTA
RGNAESRYVLTGRYDSAPATDGSGTALGWTVAWKNNYRNAHSATTW
SGQYVGGAEARINTQWLLTRGTTEANAWKSTLVGCATFTKVKPSAA
SIDAAKKAGVNNGNPLDAVQQ*

A *VWR 3510* benchtop pH Meter connected to a *Jenway* micro pH electrode or a *VWR Universal* pH electrode were used for the pH adjustment of buffers and reaction mixtures employing either 1.0 M or 0.1 M sodium hydroxide solution or hydrochloric acid.

^{1}H and ^{13}C NMR spectra were recorded in $CDCl_3$ on *Bruker Fourier 300*, *Ultrashield 400*, or *Ascend 500* instruments. Chemical shifts are reported in parts per million (ppm) and are referenced to the residual solvent resonance as the internal standard ($CHCl_3$: δ = 7.26 ppm for ^{1}H and $CDCl_3$: δ = 77.2 ppm for ^{13}C NMR).[2] Data are reported as follows: chemical shift, multiplicity (br s = broad singlet, s = singlet, d = doublet, dd = double doublet, td = triple doublet, t = triplet, dt = double triplet, q = quartet, p = pentet, sept = septet, br m = broad multiplet, m = multiplet, m_c = centrosymmetric multiplet), coupling constants (Hz) and integration.

High resolution Mass spectra (HRMS) were recorded on a *Waters LCT Premier* (ESI-(+) and APCI-(+)) or a *Waters GCT Premier* (EI) system.

Racemic samples of **S1** were obtained following a known procedure, using piperidine as catalyst. **S1** was transformed into **S2** by reduction with $NaBH_4$ in MeOH as previously reported (*vide infra*).[3]

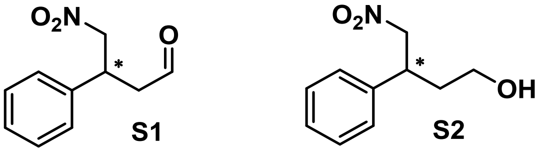

Analytical chiral HPLC analysis of product **S2** was performed on an *Agilent Technologies 1260 Infinity Quaternary* LC system using a *Phenomex Lux Cellulose-1* column, 4.6 mm × 250 mm (0.5 mL/min, 20 °C, *n*-hexane/*iso*-propanol 75:25). The absolute stereochemistry of **S2** was assigned according to Ref [3].

Size exclusion chromatography was performed using a *GE Healthcare ÄKTA Purifier* workstation or a *Bio-Rad NGC Medium-Pressure Liquid Chromatography System*.

Protein concentrations were determined using a *Thermo Scientific NanoDrop One* spectrophotometer measuring the absorption at 280 nm.

2 Experimental Details for the Preparation and Purification of T-rSav and mutants.

2.1 Expression and Purification

Tetrameric reduced streptavidin (T-rSav) and relative mutants were expressed using an *E. coli* expression system with the following protocol. Plasmid pTSA-13 containing the desired T-rSav gene in a pET-3a vector was transformed into calcium competent BL21(DE3) pLysS cells and grown for 16 h on LB agar plates containing 100 µg/mL ampicillin and 34 µg/mL chloramphenicol. A single colony from the plate was picked to inoculate a 15 mL MTP (per 1 L: 10 g tryptone, 10 g NaCl, 5 g yeast extract, 2.2 g Na_2HPO_4, 1 g KH_2PO_4, pH = 6.9) starter culture containing 100 µg/mL ampicillin and 34 µg/mL chloramphenicol, which was incubated at 37 °C and 180 rpm overnight. The culture was diluted to 40 mL with 20% glucose and then added to 1 L MTP medium containing 100 µg/mL ampicillin and 34 µg/mL chloramphenicol, yielding a final glucose concentration of 0.05%. The cultures were grown at 37 °C and 220 rpm to an OD_{600} of 1.0−1.2 and induced with IPTG at a final concentration of 1 mM. The culture was grown at 25 °C for 16 h and the cell pellet was harvested after centrifugation at 4000 rpm at 4 °C for 25 min and stored at −20 °C.

The pellet was subjected to a freeze-thaw cycle, resuspended in 25 mL of lysis buffer 1 (50 mM Tris, 100 mM NaCl, 1 mM PMSF, pH 8.0) and lysed by sonication (7 min, 5 s on, 10 s off). The insoluble fraction was isolated by centrifugation at 15000 rpm for 25 min at 4 °C. The supernatant was discarded and the insoluble fraction was washed with wash buffer 1 (4× resuspension in 50 mM Tris, 110 mM EDTA, 1.5 M NaCl, 1 mM PMSF, 0.1% Triton X-100, pH 8.0 and pellet isolation by centrifugation at 11000 rpm for 15 min and 4 °C) and wash buffer 2 (4× resuspension in 50 mM Tris, 110 mM EDTA, 1.5 M NaCl, 1 mM PMSF, pH 8.0 and pellet isolation by centrifugation at 11000 rpm for 15 min and 4 °C). The insoluble fraction was resuspended in denaturing buffer 1 (5 mL / g pellet, 6 M GdnHCl, 50 mM Tris-HCl, pH 1.5) and incubated at 37 °C and 180 rpm for 16 h. The insoluble fraction was removed by centrifugation at 15000 rpm for 25 min and 4 °C. The supernatant was diluted to 200 mL with denaturing buffer 2 (6 M GdnHCl, 50 mM Tris-HCl, pH 6.5) and dialysed against 3 L of 6 M GdnHCl, 50 mM Tris-HCl, pH 6.5 for 3 h at room temperature. The dialysis bag (3.5 kDa cut-off) was then placed into fresh 3 M GdnHCl, 50 mM Tris-HCl, pH 6.5 (denaturing buffer 2 was reused up to 5 times). T-rSav was refolded by gradient dialysis, pumping in refolding buffer (0.5 mg/L catalyst **1**, 10 mM KP_i, pH 7.0) at 4 mL/min, constant stirring and removal of the mixture at 4 mL/min for 48 h at room temperature. Towards the end of this process a varying amount of precipitation was observed. The precipitate was removed via centrifugation at 15000 rpm for 25 min at 4 °C and the supernatant was concentrated to 20 mL by *Amicon* ultra centrifugation using a 3.5 kDa cutoff. The concentrated solution was transferred into a centrifugal concentrator with a 10 kDa cutoff and the buffer was exchanged five times by concentration to 2.5 mL and refilling to 20 mL (10 mM KP_i, pH 7.0). The protein solution was finally concentrated to obtain a protein concentration of 2 mg/mL as determined by nanodrop measurement at 280 nm. This was used for catalysis of the Michael addition without further purification. A sample of the solution was loaded on SDS-PAGE to check the purity of the protein (15% w/v).

In case of purified T-rSav (WT) the concentrated protein solution obtained after *Amicon* ultra centrifugation was applied to size exclusion chromatography (*Generon ProSEC 26/60 3-70 HR* column*,* 10 mM KP_i, pH 7.0). Fractions containing protein (analysis by following 215, 255 and 280 nm UV traces) were collected and the samples loaded on SDS-PAGE to check the purity of the protein (15% w/v). Fractions containing T-rSav were pooled, transferred to a centrifugal concentrator with a 10 kDa cutoff and the buffer was exchanged to 10 mM KP_i, pH 7.0.). The protein solution was finally concentrated to obtain a protein concentration of 2 mg/mL as determined by nanodrop measurement at 280 nm.

2.2 T-rSav DNA sequence

ATGGGCATCACCGGCACCTGGTACAACCAGCTCGGCTCGACCTTCATCGTGACCGCG
GGCGCCGACGGCGCCCTGACCGGAACCTACGAGTCGGCCGTCGGCAACGCCGAGAG
CCGCTACGTCCTGACCGGTCGTTACGACAGCGCCCCGGCCACCGACGGCAGCGGCAC
CGCCCTCGGTTGGACGGTGGCCTGGAAGAATAACTACCGCAACGCCCACTCCGCGAC
CACGTGGAGCGGCCAGTACGTCGGCGGCGCCGAGGCGAGGATCAACACCCAGTGGC
TGCTGACCTCCGGCACCACCGAGGCCAACGCCTGGAAGTCCACGCTGGTCGGCCACG
ACACCTTCACCAAGGTGTAG

2.3 Site-directed mutagenesis

The Leu124, Lys121, or Ser112 mutations were introduced by site-directed mutagenesis PCR using *PrimeStar HS DNA polymerase* (*Takara*) and the accompanying buffers, dNTPs and primers mentioned in Table S1 below. Due to the high GC content of the region of interest a variety of methods and temperatures had to be screened, as primer insertions were observed, especially for mutations at Lys121. Hence a 50 µL PCR was prepared according to the instructions and the reaction mixture distributed equally (12.5 µL) over 4 PCR tubes. These were then subjected to the following conditions, using a gradient to achieve a different annealing temperature for each tube:

Method 1

Initial denaturation:	4 min, 95 °C
33 cycles:	10 s, 98 °C
	5 s, 58/60/62/64 °C
	5 min, 72 °C
Final extension:	10 min, 72 °C
Hold:	4 °C

Method 2

Initial denaturation:	4 min, 95 °C
15 cycles:	10 s, 98 °C
	5 s, 58/60/62/64 °C
	5 min, 72 °C
15 cycles:	10 s, 98 °C
	5 s, 61/63/65/67 °C
	5 min, 72 °C
Final extension:	10 min, 72 °C

Hold:	4 °C

Method 3

Initial denaturation:	4 min, 95 °C
3 cycles:	10 s, 98 °C
	5 s, 55/57/59/61 °C
	5 min, 72 °C
3 cycles:	10 s, 98 °C
	5 s, 58/60/62/64 °C
	5 min, 72 °C
30 cycles:	10 s, 98 °C
	5 s, 61/63/65/67 °C
	5 min, 72 °C
Final extension:	10 min, 72 °C
Hold:	4 °C

In case of the Lys121 mutations, Method 1 and 3 were also applied using 3% DMSO, if no positive results were obtained without DMSO. The mutant constructs were confirmed by DNA sequencing (*Eurofins Genomics*) using the T7 promoter primer.

Table S1. List of primers used for the introduction of mutations in T-rSav at positions Ser112, Lys121, and Leu124.

Mutation	Primer (5' to 3')
S112E	Forward<br>GGCTGCTGACC**GAA**GGCACCACCGAGG<br>Reverse<br>CCTCGGTGGTGCC**TTC**GGTCAGCAGCC
S112V	Forward<br>GGCTGCTGACC**GTC**GGCACCACC<br>Reverse<br>GGTGGTGCC**GAC**GGTCAGCAGCC
S112Y	Forward<br>GGCTGCTGACC**TAC**GGCACCACC<br>Reverse<br>GGTGGTGCC**GTA**GGTCAGCAGCC
K121A	Forward<br>ACCGAGGCCAACGCCTGG**GCG**TCCACGCTGGTCGGC<br>Reverse<br>GGCGTTGGCCTCGGTGGTGCCGGA
K121M	Forward<br>CGCCTGG**ATG**AGTACTCTGGTCGGCCACGACACC

	Reverse<br>GCCGACCAGAGTACT**CAT**CCAGGCGTTGGCCTCGG
K121R	Forward<br>CGCCTGG**AGG**AGTACTCTGGTCGGCCACGACACC<br>Reverse<br>GCCGACCAGAGTACT**CCT**CCAGGCGTTGGCCTCGG
L124E	Forward<br>CGTGGCCGAC**CTC**CGTGGACTTCCAGG<br>Reverse<br>CCTGGAAGTCCACG**GAG**GTCGGCCACG
L124K	Forward<br>CCTGGAAGTCCACG**AAG**GTCGGCCACG<br>Reverse<br>CGTGGCCGAC**CTT**CGTGGACTTCCAGG
L124W	Forward<br>CCTGGAAGTCCACG**TGG**GTCGGCCACG<br>Reverse<br>CGTGGCCGAC**CCA**CGTGGACTTCCAGG

3 Experimental Details for the Preparation and Purification of D-Sav and mutants.

3.1 Expression and Purification

Dimeric Streptavidin (D-Sav) and relative mutants were expressed using an *E. coli* expression system with the following protocol. Plasmid pRSF-scdSav(SARK)mv2[1] containing the desired D-Sav gene was transformed into calcium competent BL21(DE3) cells and grown for 16 h on LB agar plates containing 50 µg/mL kanamycin. A single colony from the plate was picked to inoculate a 100 mL LB starter culture containing 50 µg/mL kanamycin, which was incubated at 37 °C and 180 rpm overnight. 10 mL of the culture were added into 1 L LB medium containing 50 µg/mL kanamycin. The cultures were grown at 37 °C and 220 rpm to an OD_{600} of 0.6−0.8 and induced with IPTG at a final concentration of 1 mM. The culture was grown at 25 °C for 16 h and the cell pellet was harvested after centrifugation at 4000 rpm at 4 °C for 25 min and stored at −20 °C.

The pellet of a 1 L culture was subjected to a freeze-thaw cycle, resuspended in 50 mL of lysis buffer 2 (20 mM Tris, pH 7.4, 1 mg/mL lysozyme, 4 µg/mL DNase-I) and stirred vigorously at room temperature (20 °C) for 3 h. The soluble lysate was isolated by centrifugation at 15000 rpm for 25 min at 4 °C. The supernatant was diluted to 200 mL with denaturing buffer 2 (6 M GdnHCl, 50 mM Tris-HCl, pH 6.5) and dialysed against 3 L of 6 M GdnHCl, 50 mM Tris-HCl, pH 6.5 for 12 h at 4 °C. The dialysis bag (3.5 kDa cut-off) was then placed into 3 L of neutralising buffer (20 mM Tris, pH 7.4) for 12 h at 4 °C and finally into 3 L of imino-biotin binding buffer (50 mM $NaHCO_3$, 500 mM NaCl, pH 9.8) for 12 h at 4 °C. The lysate was subjected to centrifugation at 15000 rpm for 25 min at 4 °C. The supernatant was filtered using 0.22 µm syringe filters and the filtrate was incubated with 5 mL imino-biotin resin (*Thermo Scientific Pierce™ Iminobiotin Agarose*) for 30 min at 4 °C with gentle mixing. The resin was washed with imino-biotin binding buffer (2× 1.5 resin volume) and the protein was eluted with 1% acetic acid (5× resin volume). The elution fractions were collected in ice-cold 50 mL centrifuge tubes containing 5 mL Tris buffer (1M, pH 8.0). Samples of the wash and elution fractions were collected and run on SDS-PAGE gel (15% w/v). Elution fractions containing D-Sav were pooled and dialyzed 5 times for 12 h against 20× the volume against deionised water at 4 °C. The protein was isolated after freezing with liquid nitrogen and lyophilisation. The lyophilized D-Sav was stored at 4 °C.

3.2 D-Sav SARK DNA sequence

ATGGCAAGCATGACCGGTGGCCAGCAGATGGGTCGTGATCAGGCAGGTATTACCGGC
ACCTGGTATAATCAGCTGGGTAGCACCTTTATTGTTACCGCAGGCGCAGATGGTGCACT
GACCGGTACGTATGAAAGCGCAGTTGGTAATGCAGAAAGCCGTTATGTTCTGACAGGT
CGTTATGATAGCGCACCGGCAACCGATGGTAGCGGCACCGCACTGGGTTGGACCGTT
GCATGGAAAAATAACTATCGTAATGCACATAGCGCAACCACCTGGTCAGGTCAGTATGT
TGGTGGTGCAGAAGCACGCATTAATACCCAGTGGCTGCTGACCAGCGGCACCACCGAA
GCAAATGCCTGGGCAAGCACCCTGGTTGGTCATGATACCTTTACCAAAGTTAAACCGAG
CGCAGCAAGCATTGATGCAGCAAAAAAAGCCGGTGTGAATAATGGTAATCCGCTGGAT
GCAGTTCAGCAGGGATCCGGTGGCGGTAACGGTGGGGGAAACGGTGGCGGAAATGGT
GGAGGGAACATTGATGGTCGCGGTGGTGGTAATGCTAGCATGACTGGTGGACAGCAAA
TGGGTCGGGATCAGGCCGGCATAACCGGCACCTGGTACGCCCAGCTCGGCGATACCT
TCATCGTGACCGCGGGCGCCGACGGCGCCCTGACCGGAACCTACGTCACGGCGCGT

GGCAACGCCGAGAGCAGATACGTCCTGACCGGTCGTTACGACAGCGCCCCAGCCACC
GACGGCTCTGGCACCGCCCTCGGTTGGACGGTGGCCTGGAAGAACAATTACAGAAAC
GCCCACTCCGCGACCACGTGGAGCGGCCAATACGTCGGCGGCGCCGAGGCGAGGAT
CAACACACAATGGTTATTAACACGCGGAACTACTGAGGCCAACGCATGGAAGTCCACG
CTGGTCGGCTGCGCCACCTTCACCAAGGTGAAGCCTTCCGCCGCCTCAATCGACGCG
GCGAAGAAGGCTGGCGTCAACAACGGCAACCCTCTCGACGCCGTACAACAATAA

3.3 Site-directed mutagenesis

The Lys121$_A$ and Lys121$_B$ mutations were introduced by site-directed mutagenesis PCR using *PrimeStar HS DNA polymerase* (*Takara*) and the accompanying buffers, dNTPs and primers mentioned in Table S2 below. A 50 µL PCR was prepared according to the instructions and the reaction mixture distributed equally (12.5 µL) over 4 PCR tubes. These were then subjected Method 2 or Method 3, see 2.3.

The mutant constructs were confirmed by DNA sequencing (*Eurofins Genomics*) using the T7 promoter and terminator primers.

Table S2. List of primers used for the introduction of mutations in D-Sav at positions Lys121$_A$ and Lys121$_B$.

Mutation	Primer (5' to 3')
A121$_A$K	Forward<br>`GAAGCAAATGCCTGG`**`AAA`**`AGCACCCTGG`<br>Reverse<br>`AGGCATTTGCTTCGGTGGTGC`
K121$_B$A	Forward<br>`AGGCCAACGCATGG`**`GCG`**`TCCACGCTG`<br>Reverse<br>`TGCGTTGGCCTCAGTAGTTCCGC`

4 Experimental Details for the Preparation and Purification of M-Sav and mutants.

4.1 Expression and Purification

Monomeric streptavidin (M-Sav) and relative mutants were expressed using an *E. coli* expression system with the following protocol. Plasmid pRSET-mSA containing the desired M-Sav gene in a pRSET-A vector was transformed into calcium competent BL21 AI cells and grown for 16 h on LB agar plates containing 100 µg/mL ampicillin. A single colony from the plate was picked to inoculate a 5 mL LB culture overnight. The starter culture was diluted into 500 mL of LB medium containing 100 µg/mL ampicillin. The culture was grown at 37 °C and 225 rpm to an OD_{600} of 0.8−1.0 and induced with a final concentration of 0.5 % w/v L-arabinose. The culture was grown overnight at 20 °C. The pellet was harvested by centrifugation at 4000 rpm at 4 °C for 25 min, resuspended in 10 mL of wash buffer 3 (50 mM Tris-HCl, 100 mM NaCl, pH 8.0) and lysed via sonication (7 min, 5 s on, 10 s off). 10 mL of lysate buffer 1 (50 mM Tris-HCl, 100 mM NaCl, and 6 M GdnHCl, pH 8) were added to the suspension and left to dissolve incubating 16 hours at 4 °C. The insoluble fraction was removed by centrifugation at 4000 rpm at 4 °C for 25 min, and the supernatant was mixed with 3 mL of Ni-NTA affinity resin for six-His affinity purification. After incubation at 25 °C for 1.5 h with occasional stirring, the resin was washed twice with 1.5 volumes of resin wash buffer 1 (50 mM Tris-HCl, 100 mM NaCl, 6 M GdnHCl, and 10 mM imidazole, pH 7.5). M-Sav was eluted with elution buffer (5× resin volume, 50 mM Tris-HCl, 150 mM NaCl, 6 M GdnHCl, and 300 mM imidazole, pH 8.0). Samples of the wash and elution fractions were collected and run on SDS-PAGE gel (15% w/v). The elution fractions were added drop by drop to 5 times their total volume of ice-cold refolding buffer 2 (50 mM Tris-HCl, 150 mM NaCl, 0.3 mg/mL catalyst **1**, **2**, or biotin, 0.2 mg/mL oxidized glutathione, and 1 mg/mL reduced glutathione) under rapid stirring to refold the protein by stirring overnight. The precipitates were removed by centrifugation at 15000 rpm at 4 °C for 25 min. The refolded protein solution was concentrated to 5 mL using *Amicon* ultra centrifugation with a 10 kDa cut-off. The concentrated protein solution was applied to size exclusion chromatography (*Hi-Load*™ *16/600 Superdex 200 pg*, 50 mM Tris-HCl, 150 mM NaCl, pH 8.0). Fractions containing protein (analysis by following 215, 255 and 280 nm UV traces) were collected and the samples loaded on SDS-PAGE to check the purity of the protein (15% w/v). Fractions containing M-Sav were pooled, transferred to a centrifugal concentrator with a 10 kDa cut-off and the buffer was exchanged to the respective buffer (see 5.2 and see 14.1, Tables S8–S10). The protein solution was finally concentrated to obtain a protein concentration of 2 mg/mL as determined by nanodrop measurement at 280 nm.

4.2 M-Sav DNA sequence

ATGGGCAGCAGCCATCACCATCATCACCACAGCCAGGATCTGGCTAGCGCGGAAGCG
GGTATCACCGGCACGTGGTACAACCAGTCTGGTTCTACCTTCACCGTTACCGCGGGTG
CGGACGGTAACCTGACCGGTCAGTACGAAAACCGTGCGCAGGGCACTGGTTGCCAGA
ACTCTCCGTACACCCTGACCGGTCGTTACAACGGTACCAAACTGGAATGGCGTGTTGA
ATGGAACAACTCTACCGAAAACTGCCACTCTCGTACCGAATGGCGTGGTCAGTACCAG
GGTGGTGCGGAAGCGCGTATCAACACCCAGTGGAACCTGACCTACGAAGGTGGTTCT

GGTCCGGCGACCGAACAGGGTCAGGACACCTTCACCAAAGTTAAACCGTCTGCGGCG
TCTGGATCCGACTACAAGGACGATGACGACAAGTAA

4.3 Site-directed mutagenesis

The mutations were introduced by site-directed mutagenesis PCR using *PrimeStar HS DNA polymerase* (*Takara*) and the accompanying buffers, dNTPs and primers mentioned in Table S3 below. For single mutants at Y111 the mentioned forward primers are used in conjunction with reverse primer GGTCAGGTTCCACTGGTGTTG. For all single mutants, the following protocol was used:

Method 4

Initial denaturation:	3 min, 95 °C
33 cycles:	10 s, 98 °C
	5 s, 63 °C
	4 min, 72 °C
Final extension:	10 min, 72 °C
Hold:	4 °C

The double mutant (Y111S E120L) was obtained by preparing a 50 µL PCR setup according to the instructions and equal distribution of the reaction mixture (12.5 µL) over 4 PCR tubes for an annealing temperature screen according to **Method 2**. The mutant constructs were confirmed by DNA sequencing (*Eurofins Genomics*) using the T7 promoter primer.

Table S3. List of primers used for the introduction of mutations in M-Sav at positions Y111, and E120.

Mutation	Primer (5' to 3')
Y111A	Forward<br>CAGTGGAACCTGACC**GCG**GAAGGTGGTTCTGGTCCGGCGACCGAAC<br>Reverse<br>GGTCAGGTTCCACTGGTGTTG
Y111K	Forward<br>CAGTGGAACCTGACC**AAA**GAAGGTGGTTCTGGTCCGGCGACCGAAC
Y111S	Forward<br>CAGTGGAACCTGACC**AGC**GAAGGTGGTTCTGGTCCGGCGACCGAAC
Y111T	Forward<br>CAGTGGAACCTGACC**ACC**GAAGGTGGTTCTGGTCCGGCGACCGAAC
Y111V	Forward<br>CAGTGGAACCTGACC**GUG**GAAGGTGGTTCTGGTCCGGCGACCGAAC
E120L	Forward<br>TCCGGCGACC**CTG**CAGGGTCAGGACACC<br>Reverse

	GGTGTCCTGACCCTG**CAG**GGTCGCCGGA
Y111S E120L	Forward<br>AGTGGAACCTGACC**AGC**GAAGGTGGTTCTGGTCCGGCGACC**CTG**CAGGGTCAGG<br>ACACCTTCACCAAAGTTAAACCGTCTGCGG<br>Reverse<br>GTGTCCTGACCCTG**CAG**GGTCGCCGGACCAGAACCACCTTC**GCT**<br>GGTCAGGTTCCACTGGGTGTTGATACGCGCTTCCGCACC

4.4 Circular Dichroism experiments

The circular dichroism spectra of M-Sav were recorded on a *Chirascan*^{TM} *CD spectrophotometer* (*Applied Photophysics*) with a temperature controller using a cuvette with a path length of 1 mm. A spectral bandwidth of 1 nm was used for data collection. M-Sav was dissolved in PBS buffer (pH 7.4) at a concentration of 50 µM using 500 µM of the appropriate ligand. The blank measurement was prepared using 500 µM of the appropriate ligand or just using the buffer. To induce heat denaturation, the temperature was increased from 4 to 96 °C, and the CD spectra were recorded between 200 and 400 nm for every 2 °C increment with 1 nm steps. Melting temperatures were checked at 234 nm.

5 Experimental Details for the Activity and Selectivity Screening of Sav-based Hybrid Catalysts in the Michael Addition of Nitromethane to Cinnamaldehyde

5.1 ^{1}H NMR Based Screening for Yield – T-Sav

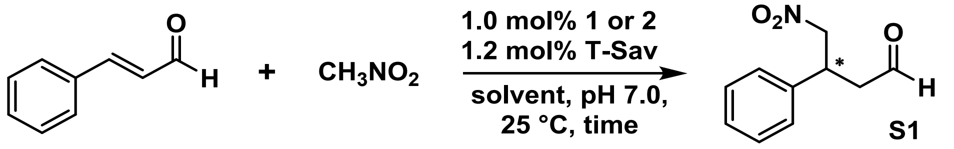

Runs in KP$_i$ Buffer

A stock solution of the respective biotinylated organocatalyst **1** or **2** (2.59 mM in KP$_i$ buffer, pH 7.0, 10 mM) was prepared. 0.58 mg commercial T-Sav (38 nmol active sites, 1.2 mol%, contains 75 µg KP$_i$ mixture, pH 6.5) were weighed into a 1.5 mL Microcentrifuge tube. 400 µL of buffer (KP$_i$ buffer, pH 7.0, 10 mM) as well as 12 µL of the organocatalyst stock solution (31 nmol, 1.0 mol%) were added to T-Sav. The suspension was mixed by inversion, spun down in a microcentrifuge tube centrifuge (13,000 *g*), and the pH was adjusted carefully to 7.0 by using 0.1 M NaOH and 0.1 M HCl stock solutions. The mixture was filled up to 500 µL with buffer (KP$_i$ buffer, pH 7.0, 10 mM). Stock solutions of cinnamaldehyde (1.33 M in MeOH, HPLC grade) and nitromethane (6.61 M in MeOH, HPLC grade) were prepared. Subsequently, 2.5 µL of the nitromethane stock solution (16.5 µmol, 5.0 eq) and 2.5 µL of the aldehyde stock solution (3.3 µmol, 1.0 eq) were added to the catalyst solution. The microcentrifuge tube was placed in a Falcon tube, which was placed inside an incubator shaker (50 rpm, 25 °C) for 42 h. The tube was taken out of the incubator and the aqueous mixture was extracted once with $CDCl_3$ (700 µL). The organic phase was transferred into an NMR tube and directly subjected to ^{1}H NMR analysis.

Runs in KP$_i$ Buffer / MeOH

A stock solution of the respective biotinylated organocatalyst **1** or **2** (2.59 mM in KP$_i$ buffer, pH 7.0, 10 mM) was prepared. 0.58 mg commercial T-Sav (38 nmol active sites, 1.2 mol%, contains 75 µg KP$_i$ mixture, pH 6.5) were weighed into a 1.5 mL Microcentrifuge tube. 200 µL of buffer (KP$_i$ buffer, pH 7.0, 10 mM) as well as 12 µL of the organocatalyst stock solution (31 nmol, 1.0 mol%) were added to T-Sav. The suspension was mixed by inversion, spun down in a microcentrifuge tube centrifuge (13,000 g), and the pH was adjusted carefully to 7.0 by using 0.1 M NaOH and 0.1 M HCl stock solutions. The mixture was filled up to 250 µL with buffer (KP$_i$ buffer, pH 7.0, 10 mM). Stock solutions of cinnamaldehyde (1.33 M in MeOH, HPLC grade) and nitromethane (6.61 M in MeOH, HPLC grade) were prepared. Subsequently, 2.5 µL of the nitromethane stock solution (16.5 µmol, 5.0 eq) and 2.5 µL of the aldehyde stock solution (3.3 µmol, 1.0 eq) were added to the catalyst solution. 250 µL of MeOH were added. The microcentrifuge tube was placed in a Falcon tube, which was placed inside an incubator shaker (50 rpm, 25 °C) for 18 h. The phases were separated, the aqueous phase was extracted (3 × 500 µL EtOAc, 3 × 500 µL $CHCl_3$), and the organic phases were combined. The volatiles were removed under reduced pressure, the residue was taken up in 700 µL $CDCl_3$, and transferred to an NMR tube. This was directly subjected to ^{1}H NMR analysis.

Catalyst Background Runs

To determine the background reactivity of **1** and **2** reactions were performed exactly as stated above without the use of T-Sav.

5.2 ^{1}H NMR Based Screening for Yield – T-rSav

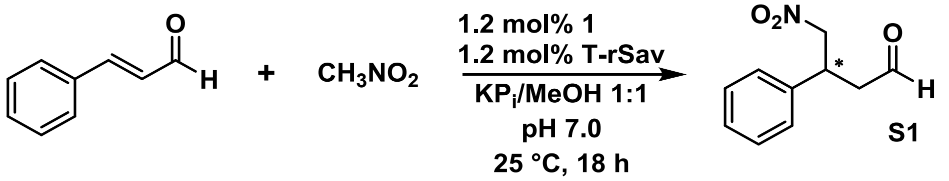

General Procedure for all Runs

Stock solutions of cinnamaldehyde (1.33 M in MeOH, HPLC grade) and nitromethane (6.61 M in MeOH, HPLC grade) were prepared. 250 µL of the T-rSav:**1** solution obtained from 2.1 were added into a 1.5 mL Eppendorf tube (39 nmol, 1.2 mol %). 2.5 µL of the nitromethane stock solution (16.5 µmol, 5.0 eq) and 2.5 µL of the aldehyde stock solution (3.3 µmol, 1.0 eq) were added to the catalyst solution. 250 µL of MeOH were added. The microcentrifuge tube was placed in a Falcon tube, which was placed inside an incubator shaker (50 rpm, 25 °C) for 18 h. The phases were separated, the aqueous phase was extracted (3 × 500 µL EtOAc, 3 × 500 µL $CHCl_3$), and the organic phases were combined. The volatiles were removed under reduced pressure, the residue was taken up in 700 µL $CDCl_3$, and transferred to an NMR tube. This was directly subjected to ^1H NMR analysis.

5.3 ^{1}H NMR Based Screening for Yield – D-Sav

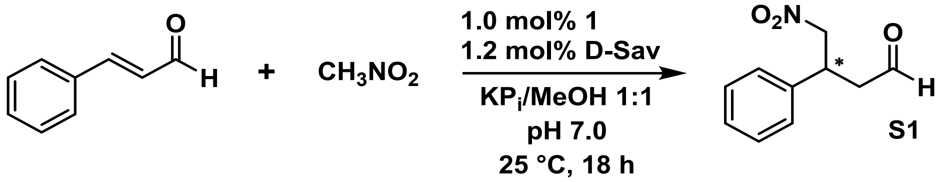

General Procedure for all Runs

A stock solution of the respective biotinylated organocatalyst **1** or **2** (2.59 mM in KP_i buffer, pH 7.0, 10 mM) was prepared. At least 1.33 mg of lyophilized D-Sav from 3.1 (38 nmol active sites, 1.2 mol%) were weighed into a 1.5 mL Microcentrifuge tube. 238 µL of buffer (KP_i buffer, pH 7.0, 10 mM) as well as 12 µL of the organocatalyst stock solution (31 nmol, 1.0 mol%) were added to D-Sav. The suspension was mixed by inversion and spun down in a microcentrifuge tube centrifuge (13,000 g). Stock solutions of cinnamaldehyde (1.33 M in MeOH, HPLC grade) and nitromethane (6.61 M in MeOH, HPLC grade) were prepared. Subsequently, 2.5 µL of the nitromethane stock solution (16.5 µmol, 5.0 eq) and 2.5 µL of the aldehyde stock solution (3.3 µmol, 1.0 eq) were added to the catalyst solution. 250 µL of MeOH were added. The microcentrifuge tube was placed in a Falcon tube, which was placed inside an incubator shaker (50 rpm, 25 °C) for 18 h. The phases were separated, the aqueous phase was extracted (3 × 500 µL EtOAc, 3 × 500 µL $CHCl_3$), and the organic phases were combined. The volatiles were removed under reduced pressure, the residue was taken up in 700 µL $CDCl_3$, and transferred to an NMR tube. This was directly subjected to ^1H NMR analysis.

5.4 ^{1}H NMR Based Screening for Yield – M-Sav

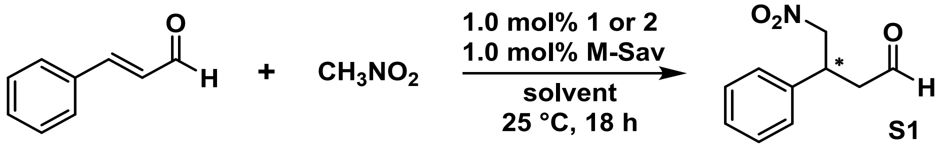

General Procedure for all Runs

Stock solutions of cinnamaldehyde (1.33 M in MeOH, HPLC grade) and nitromethane (6.61 M in MeOH, HPLC grade) were prepared. 250 µL of the respective (see 14.1, Tables S8–S10) M-Sav solution obtained from 4.1 were added into a 1.5 mL Eppendorf tube (32 nmol, 1 mol% %). To the solution were added aliquots of the nitromethane (5.0 µL, 33 µmol, 10.0 eq) and cinnamaldehyde (2.5 µL, 3.3 µmol, 1.0 eq) stock solutions, and the respective buffer and organic solvent (see 14.1, Tables S8–S10). The mixture was homogenized by shaking at 500 rpm for 5 min at 25 °C and the mixture was shaken at 300 rpm at 25 °C for 18 h. CH_2Cl_2 (500 µL) was added, the biphasic mixture was shaken vigorously for 1 min, and the organic phase was isolated. This operation was repeated three times, the organic phases were pooled, and evaporated under reduced vacuum. The crude of reaction was dissolved in $CDCl_3$ and subjected to ^{1}H NMR analysis.

5.5 ^{1}H NMR Based Screening for Yield – Exemplary Analysis

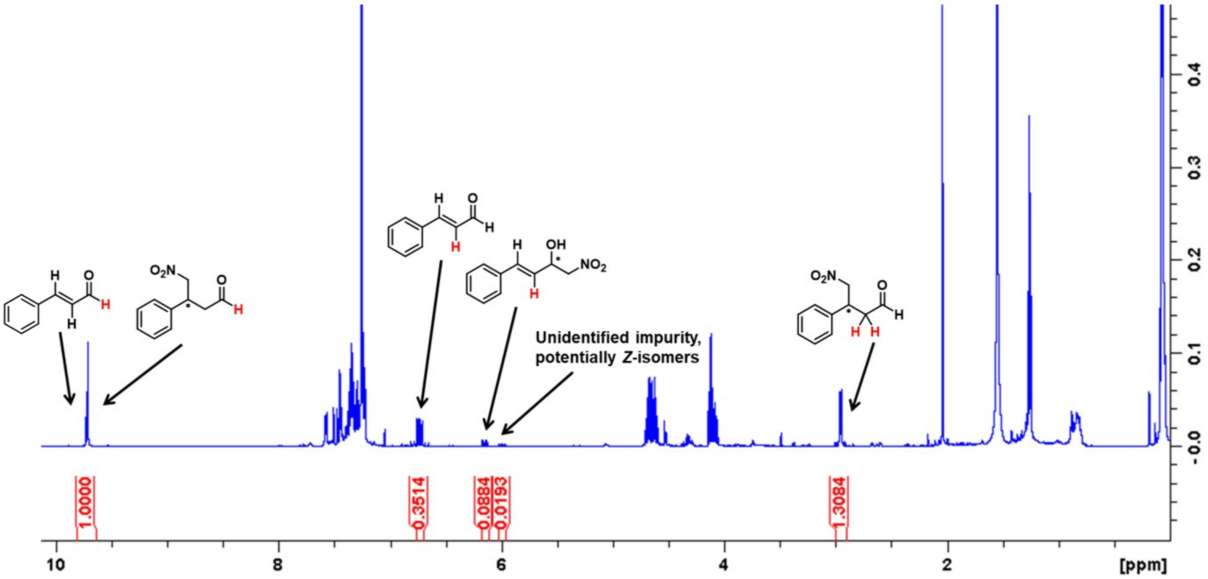

Figure S1. ^{1}H NMR Based Screening for Yield – Exemplary Analysis. Yields were calculated as followed using the above indicated integrals of the starting material cinnamaldehyde, the 1,4-addition product, the 1,2-addition product,[4] and an unidentified impurity.

$$\text{Yield 1,4-addition product} = \frac{1 - 0.3514 + \frac{1.3084}{2}}{2} \times \frac{100\%}{1 + 0.0884 + 0.0193} = 59\%$$

Yield 1,2-addition product: $\frac{0.0884 \times 100\%}{1 + 0.0884 + 0.0193} = 8\%$

Conversion: 59% + 8% = 67%

5.6 Enantioselectivity Determination for all Sav Variants

After determining the conversion by ^{1}H NMR, the samples of each triplicate were combined, and the solvent was removed. The crude material was purified by preparative TLC (*n*-hexane:EtOAc 75:25) using a complete sheet. The part containing **S1** (checked via racemic reference sample by UV fluorescence deletion and permanganate stain) was cut out, the silica scratched from the aluminium plate, and stirred in CH_2Cl_2 for several minutes. The silica was filtered off, washed with CH_2Cl_2, the filtrate was concentrated under reduced pressure, and the purified product was stored at –23 °C under inert atmosphere until further usage

The purified compound **S1** was converted to alcohol **S2** according to [3] via reduction with $NaBH_4$, using MeOH as solvent instead of ethanol. The crude material obtained according to Ref [3] was purified by preparative TLC (*n*-hexane:EtOAc 66:33) using a complete sheet. The part containing **S2** (checked via racemic reference sample by UV fluorescence deletion and permanganate stain) was cut out, the silica scratched from the aluminium plate, and stirred in CH_2Cl_2 for several minutes. The silica was filtered off, washed with CH_2Cl_2, the filtrate was concentrated under reduced pressure, and the purified product was used for analytical chiral HPLC to determine the enantioselectivity of the respective catalyst.

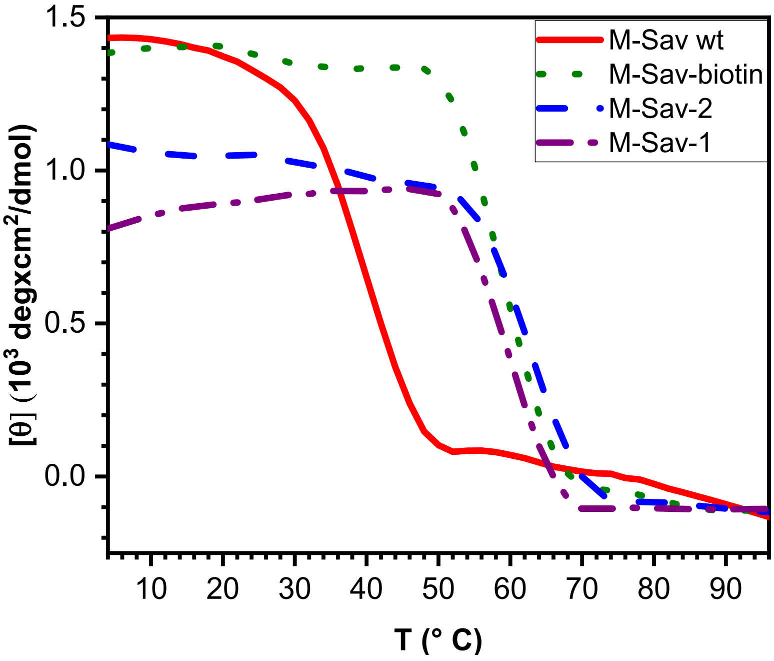

Host-Ligand	T_m / °C
M-Sav	37.3 ± 2.7
M-Sav:biotin	59.1 ± 0.5
M-Sav:1	63.1 ± 0.1
M-Sav:2	62.9 ± 0.5

Figure S2. Melting Temperatures of M-Sav alone and in the presence of biotinylated catalyst 1 and 2. (See **Section 12** for CD spectra)

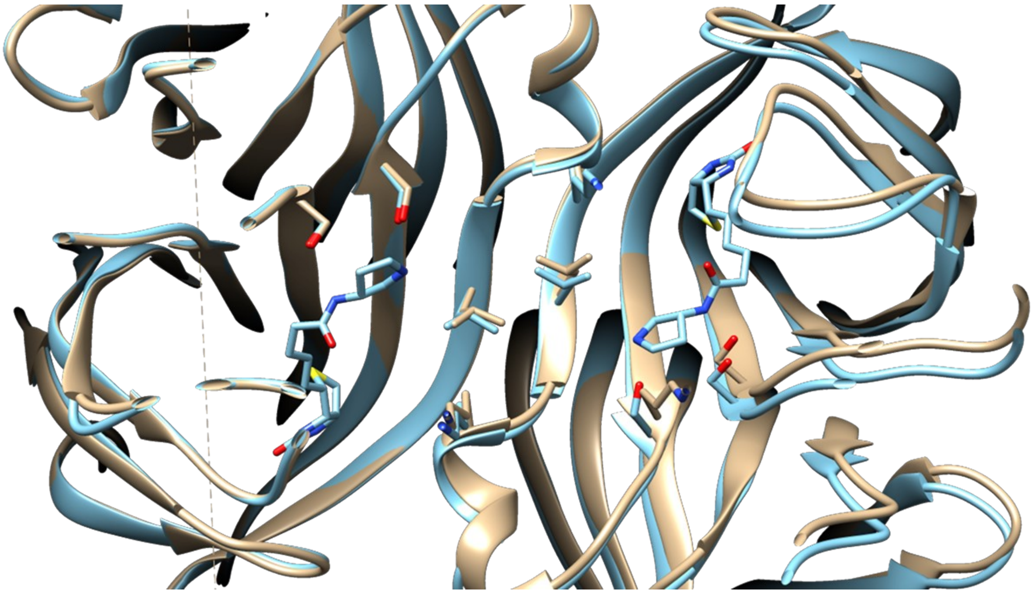

Figure S3. Overlay of T-Sav:1 (light grey, PDB: 6GH7) and D-Sav (light brown, PDB: 6S50). Ligands of D-Sav and the two subunits in the back are omitted for clarity.

6 Experimental Details for the Structure Determination of T-Sav:2

6.1 Procedure for Crystallization of T-Sav:2

Crystals of T-Sav:**2** were grown by adapting protocols for the preparation of T-Sav:**1** crystals. A solution of commercial T-Sav (*Prospec* 791, 10 mg/mL) was incubated with **2** (5 equivalents per active biotin binding site) for 10 minutes. Residual KP_i from the commercial lyophilised protein was removed by buffer exchange via spin column (10k MWCO) to produce a solution of T-Sav:**2** in deionised water (40 mg/mL). This was used as protein stock solution to prepare samples for crystallisation.

The crystallisation experiments were performed in a 24-well plate employing 4 different conditions in each quadrant of 6 wells, with slightly varied conditions within the quadrants (see Table S4). All crystals were grown using the hanging-drop vapour-diffusion method. Each well was filled with 1 mL of the precipitant solution (Table S4). On the respective cover slip, three drops were produced by mixing the protein solution (1, 2, and 3 µL) with the same volume of precipitant solution. The assay was stored in an incubator at 20 °C and the crystals appeared within 10 days.

Table S4. Overview of crystallization conditions used in the 24 well plate.

Precipitant solution	mg/mL protein, % PEG 8000			Precipitant solution	mg/mL protein, % $(NH_4)_2SO_4$		
0.1 M HEPES, pH 7.5	25, 15	25, 16	25, 17	0.1 M NaOAc, pH 4.5, 0.2 M NaCl	26, 30	26, 35	26, 40
	30, 15	30, 16	30, 17		35, 30	35, 35	35, 40
Precipitant solution	mg/mL protein, % $(NH_4)_2SO_4$			Precipitant solution	mg/mL protein, % MPD		
5% 2-Propanol	12.5, 55	12.5, 60	12.5, 65	Deionized water	25, 49	25, 52	25, 55
	25, 55	25, 60	25, 65		30, 49	30, 52	30, 55

6.2 X-ray data collection, processing and structure solution

Crystals grown from various drops were fished and flash-frozen with liquid nitrogen. Diffraction data were collected at 100 K at beamlines I04 of the Diamond Light Source, Didcot, UK, with all images integrated with XDS and scaled with Aimless in xia2. The crystals grown in 52% MPD gave the best resolution. The structures were solved by molecular replacement using MOLREP in CCP4i2, using PDB 6GH7 as a search model. Model was refined numerous cycles with maximum likelihood refinement using REFMAC and manually corrected using COOT.

6.3 Crystallographic Details for the Obtained Crystal Structure of T-Sav:2

Table S5 Data collection and refinement statistics by Molecular replacement

	T-Sav:2
**PDB code**	7NLV
**Data collection**	
Space group	$P\ 1\ 2_1\ 1$
Cell dimensions	
a, *b*, *c* (Å)	49.32, 97.60, 50.90
α, β, γ (°)	90.00, 110.26, 90.00
Resolution (Å)	46.27 (1.29)
R_{merge}	0.052 (0.490)
$I/\sigma I$	7.3 (1.4)
Completeness (%)	99.5 (99.9)
Redundancy	1.9 (1.9)
**Refinement**	
Resolution (Å)	1.29
No. reflections	112639/5708
$R_{work/}$ R_{free}	0.207/0.247
No. atoms	
Protein	3592
Ligand/ion	84
Water	280
B-factors	
Protein	24.96
Ligand/ion	21.9
Water	38.15
R.m.s deviations	
Bond lengths (Å)	0.0097
Bond angles (º)	1.603

*Highest resolution shell is shown in parenthesis.

7 Experimental Details for the Structure Determination of M-Sav:2

7.1 Procedure for Crystallization of M-Sav:2

Stock solutions of M-Sav:**2** (20 mM Tris, 50 mM NaCl, pH 8.0) were obtained by the protocol of Chapter 3.1 but applying a second size exclusion chromatography run to obtain highly pure material. Crystals of M-Sav:**2** were grown in 96 well crystallization plates by employing a crystallization robot and a commercial screen (*Molecular Dimensions*, *Structure Screen 1 + 2 HT-96 Single Reagent*) using the sitting drop method. The assay was stored in an incubator at 20 °C.

7.2 X-ray data collection, processing and structure solution

Crystals grown from various drops were fished and flash-frozen with liquid nitrogen. Diffraction data were collected at 100 K at beamlines I03 of the Diamond Light Source, Didcot, UK, with all images integrated with XDS and scaled with Aimless in xia2. The crystals grown in 0.2 M $(NH_4)_2SO_4$, 0.1 M $Na(CH_3)_2AsO_2$, 30% w/v PEG 8000, pH 6.5 gave the best resolution. The structures were solved by molecular replacement using MOLREP in CCP4i2, using PDB 4JNJ as a search model. Model was refined numerous cycles with maximum likelihood refinement using REFMAC and manually corrected using COOT.

7.3 Crystallographic Details for the Obtained Crystal Structure of M-Sav:2

Table S6 Data collection and refinement statistics by Molecular replacement

	M-Sav:2
**PDB code**	6ZYT
**Data collection**	
Space group	P_1
Cell dimensions	
a, *b*, *c* (Å)	41.79, 52.74, 89.36
α, β, γ (°)	75.24, 78.24, 84.88
Resolution (Å)	25.48 (1.80) *
R_{merge}	0.095 (0.367)
$I/\sigma I$	5.6 (1.63)
Completeness (%)	95.7 (95.0)
Redundancy	1.7 (1.7)
**Refinement**	
Resolution (Å)	1.80
No. reflections	111781/6396
$R_{work/}$ R_{free}	0.262/0.306
No. atoms	
Protein	5231
Ligand/ion	105/15
Water	250
B-factors	
Protein	37.17
Ligand/ion	30.0/45.3
Water	37.8
R.m.s deviations	
Bond lengths (Å)	0.0146
Bond angles (º)	2.097

*Highest resolution shell is shown in parenthesis.

8 Computational Methods

8.1 Computational Details of the Molecular Models Set Up

The origin of atom coordinates of streptavidin with bound biotin was adapted from X-Ray structure as available in Protein Data Bank (PDB 1STP).[5] Biological assembly, missing atoms, protonation state of titratable amino acids, optimization and molecular dynamics (MD) simulations performed for the T-Sav with biotin-catalyst were described in our previous paper.[3] The last structure of these MD simulations was used in order to build up the model to study the molecular mechanism of the iminium catalysis based on Quantum Mechanics/Molecular Mechanics (QM/MM) methods. After optimization of the system, those residues located 20 Å far away from any of the substrate atoms were kept frozen in the remaining calculations. Potential energy surfaces, free energy surfaces and spline corrections have been performed using *fDynamo* library[6] together with the OPLS force field.[7] A cut-off for non-bonding interactions was applied using a smooth switching function between 14.5 Å to 16 Å. Parameters for parts of the catalyst included in the MM part were generated using the *SwissParam* web server.[8]

Additionally, two new models have been prepared to study the mutations of the residues Lys121 (Lys121Ala) and Ser112 (Ser112Ala) by Ala. MD simulations were performed at MM level using NAMD software.[9] The behaviour of the system was controlled by using AMBER ff-03 parameters.[10] NPT MD of 5 ns for Lys121Ala and Ser112Ala models with time step of 1 fs at 300 K were carried out after previous optimization, heating (from 0 to 300 K with 0.001 K temperature increment) and equilibration processes of 100ps. The constant temperature and pressure were controlled using the Langevin piston method.[11] Periodic boundary conditions (PBC) using the particle mesh Ewald method were applied. A Cut-off for nonbonding interactions was applied using a smooth switching function with between 14.5 to 16 Å.

8.2 Computational details of the QM/MM simulations

In this work, an additive hybrid QM/MM scheme was employed for the construction of the total Hamiltonian, $\hat{H}_{eff}$, where the total energy is obtained from the sum of each contribution to the energy.

$$\hat{H}_{eff} = \hat{H}_{QM} + \hat{H}_{QM/MM}^{elec} + \hat{H}_{QM/MM}^{VdW} + \hat{H}_{MM} \quad \text{(S1)}$$

Here, $\hat{H}_{QM}$ describes the atoms in the QM part, $\hat{H}_{QM/MM}$ defines the interaction between the QM and MM region and $\hat{H}_{MM}$ describes the rest of the MM part. As shown in Scheme S1, the QM subset of atoms includes part of the biotin catalyst, cinamaldehyde, nitromethane, a molecule of water and part of $Lys121_B$.

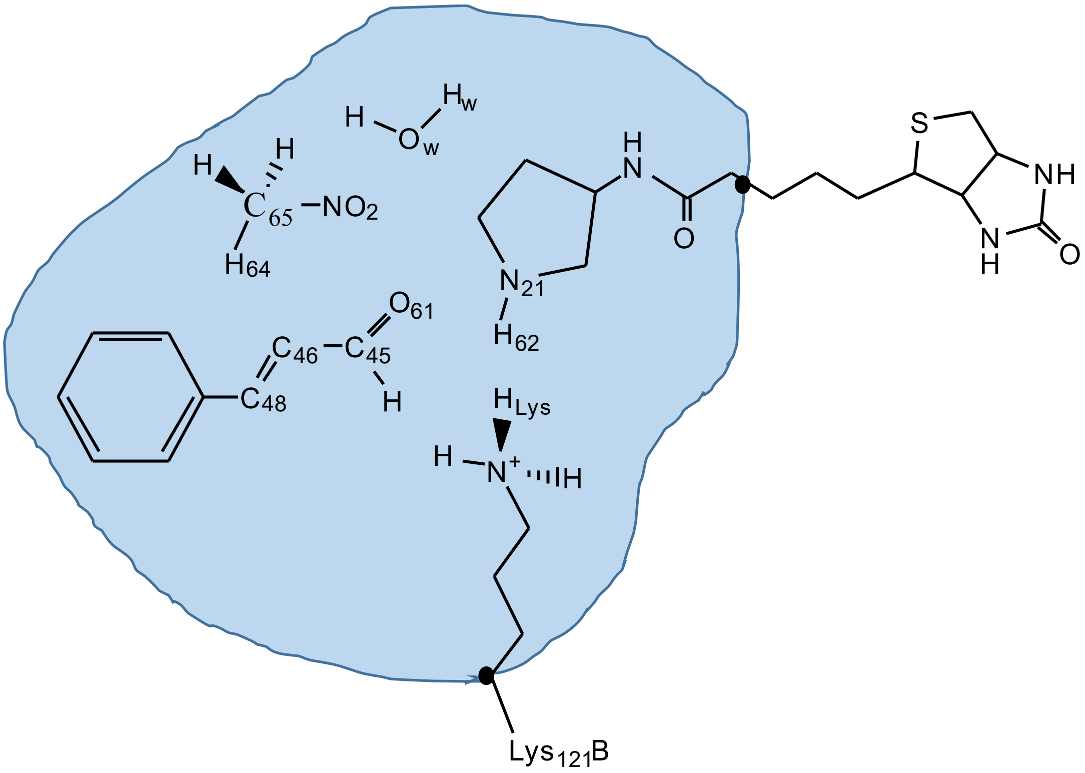

Figure S4. Schematic representation of the active site of T-Sav. QM region is represented as blue region. Black dots represent link atoms between QM and MM region. Key atoms involved in the reaction are labelled

8.3 Potential Energy Surfaces

Exploration of the Potential Energy Surfaces (PES) was carried out by choosing the appropriate combination of internal coordinates (ξ_i) in every single step of the reaction. A harmonic constraint was used to maintain the proper interatomic distances along the reaction coordinate, and a series of conjugate gradient optimizations and L-BFGS-B optimization algorithms were applied to obtain the final potential energy of the minimized constrained geometry. The QM sub-set of atoms were described first by the Austin Model 1 (AM1)[12] semi-empirical Hamiltonian.
After the exploration of stationary points on the PES, the structures corresponding to reactants and products states (RS and PS, respectively), intermediates (Is) and transition state (TSs) were localized applying Baker's algorithm.[13] Minimum energy path was traced down to reactants and products following the Intrinsic Reaction Coordinate (IRC) method from every localized TS structure. The QM sub-set of atoms in these energy minimization were treated by the AM1 Hamiltonian and later by the M06-2X functional[14] with the standard 6-31+G(d,p) basis set using the Gaussian09 program.[15]

8.4 Free Energy Surfaces

FESs were obtained, in terms of Potentials of Mean Force (1D- and 2D-PMF), for every step of the reaction using the Umbrella Sampling approach[16] combined with the Weighted Histogram Analysis Method (WHAM).[17] Series of MD simulations were performed adding a constraint along the selected reaction coordinates with an umbrella force constant of 2500 $kJ\cdot mol^{-1}\cdot Å^{-2}$. In every window QM/MM MD simulations were performed with a total of 5 ps of equilibration and 20 ps of production at 303 K using the Langevin-Verlet algorithm with a time step of 1 fs. Structures obtained in previously computed PESs were used as starting points for the MD simulations in every window. The resulting Free Energy Surfaces are shown in Figures S5A-S5C while average of key inter-atomic distances of every state involved in the reaction are listed in Table S7.

8.5 Spline Corrections

A correction term is interpolated to any value along the reaction coordinates in the FES. A continuous energy function is used to obtain the corrected PMFs:

$$E = E_{LL/MM} + S[\Delta E_{LL}^{HL}(\xi_1,\xi_2)] \qquad \text{(S2)}$$

where *S* is the two-dimensional spline function and ΔE_{LL}^{HL} is the difference between the energies obtained at low-level (LL) and high-level (HL) of theory of the QM part. The AM1 semi-empirical Hamiltonian was used as LL method, while a density functional theory (DFT)-based method was selected for the HL energy calculation. In particular, HL energy calculations were performed by means of the hybrid M06-2X[11] functional using the standard 6-31+G(d,p) basis set. These calculations were carried out using the Gaussian09 program.

8.6 Computational Results

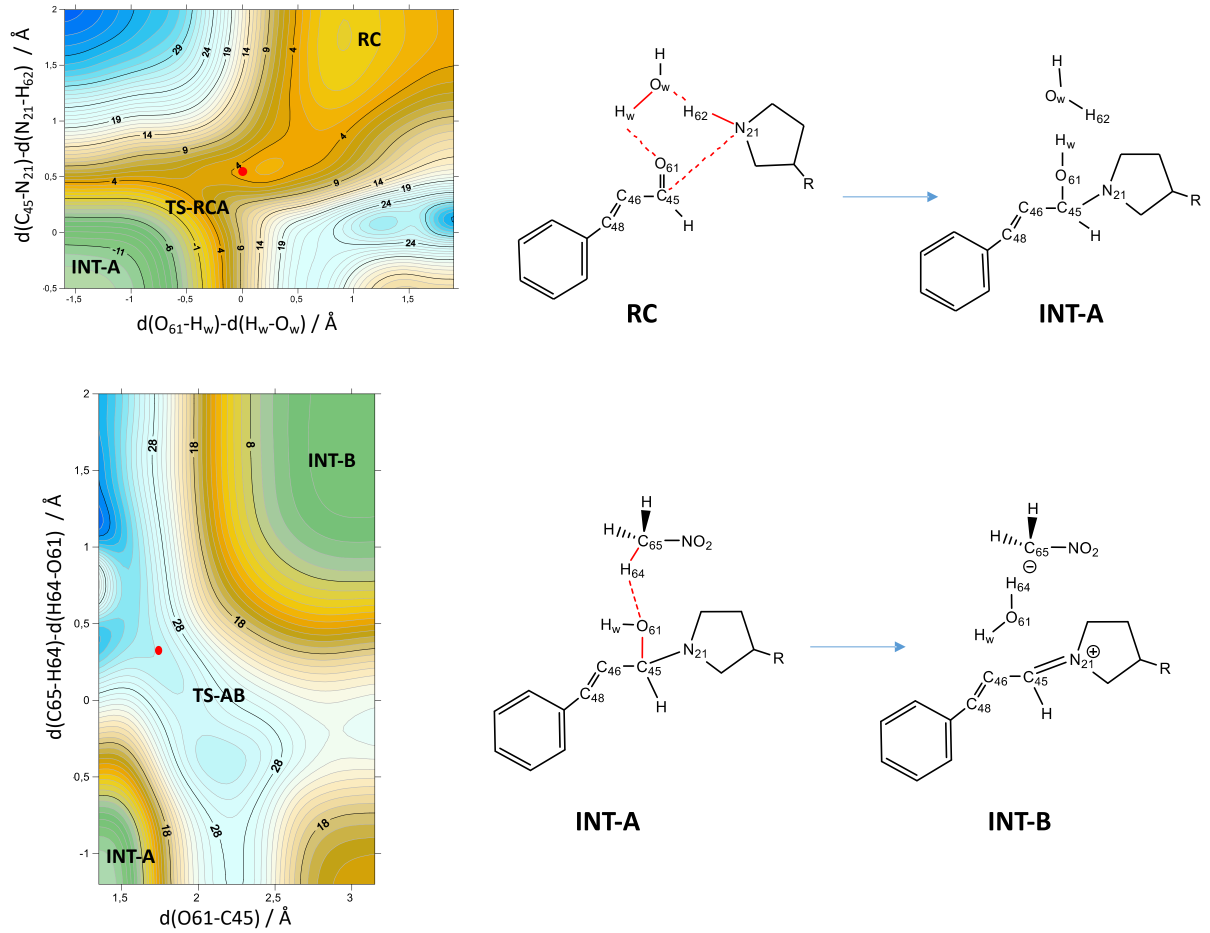

Figure S5a: Free energy surfaces of the RS to INT-A and INT-A to INT-B chemical steps of the organocatalytic reaction hosted by the T-Sav computed computed in terms of 2D-PMFs at M06-2X:AM1/MM level. Distances are in Å and energies of isoenergetic lines in kcal·mol^{-1}. Position of TSs structures optimized at M06-2X/MM level are indicated as red dots on the surfaces. Schematic representations of every chemical step, indicating in red those bonds that are being formed and/or broken.

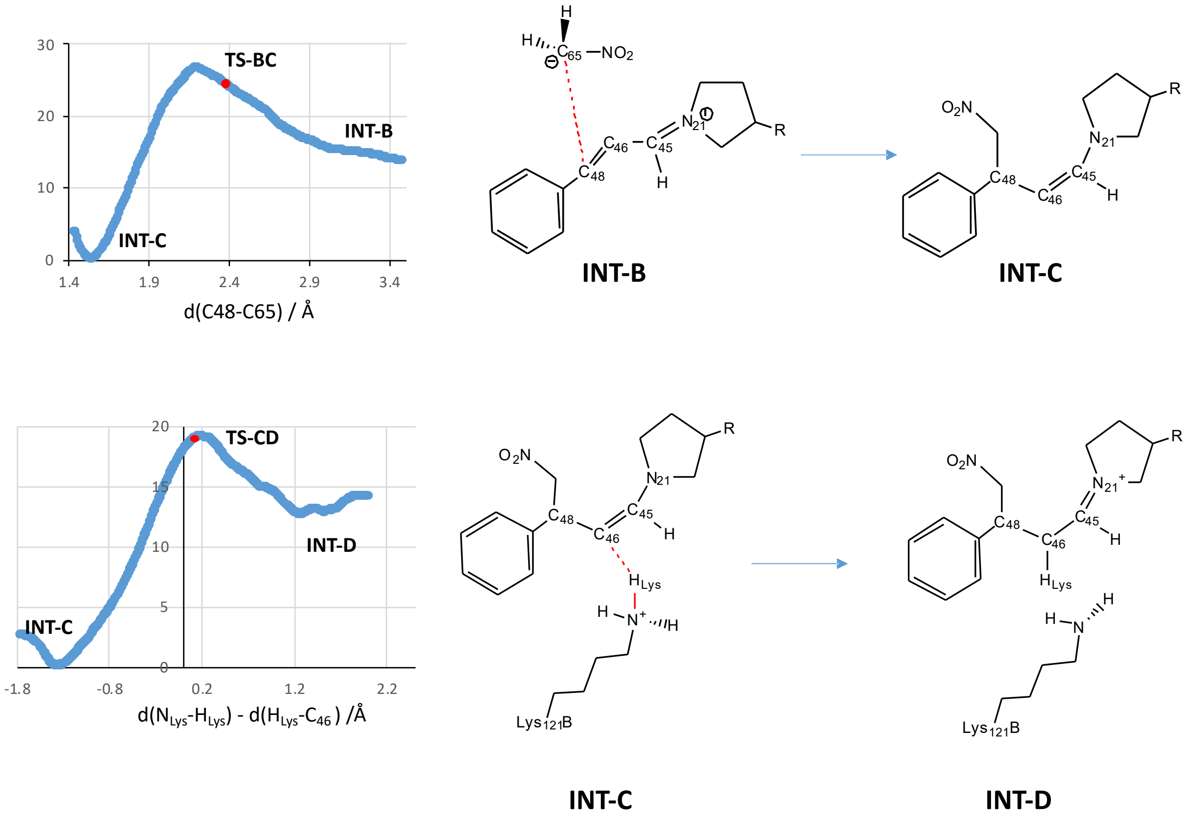

Figure S5b. Free energy surfaces of the INT-B to INT-C and INT-C to INT-D chemical steps of the organocatalytic reaction hosted by the T-Sav computed computed in terms of 1D-PMFs at M06-2X:AM1/MM level. Distances are in Å and energies of isoenergetic lines in kcal·mol^{-1}. Position of TSs structures optimized at M06-2X/MM level are indicated as red dots on the surfaces. Schematic representations of every chemical step, indicating in red those bonds that are being formed and/or broken.

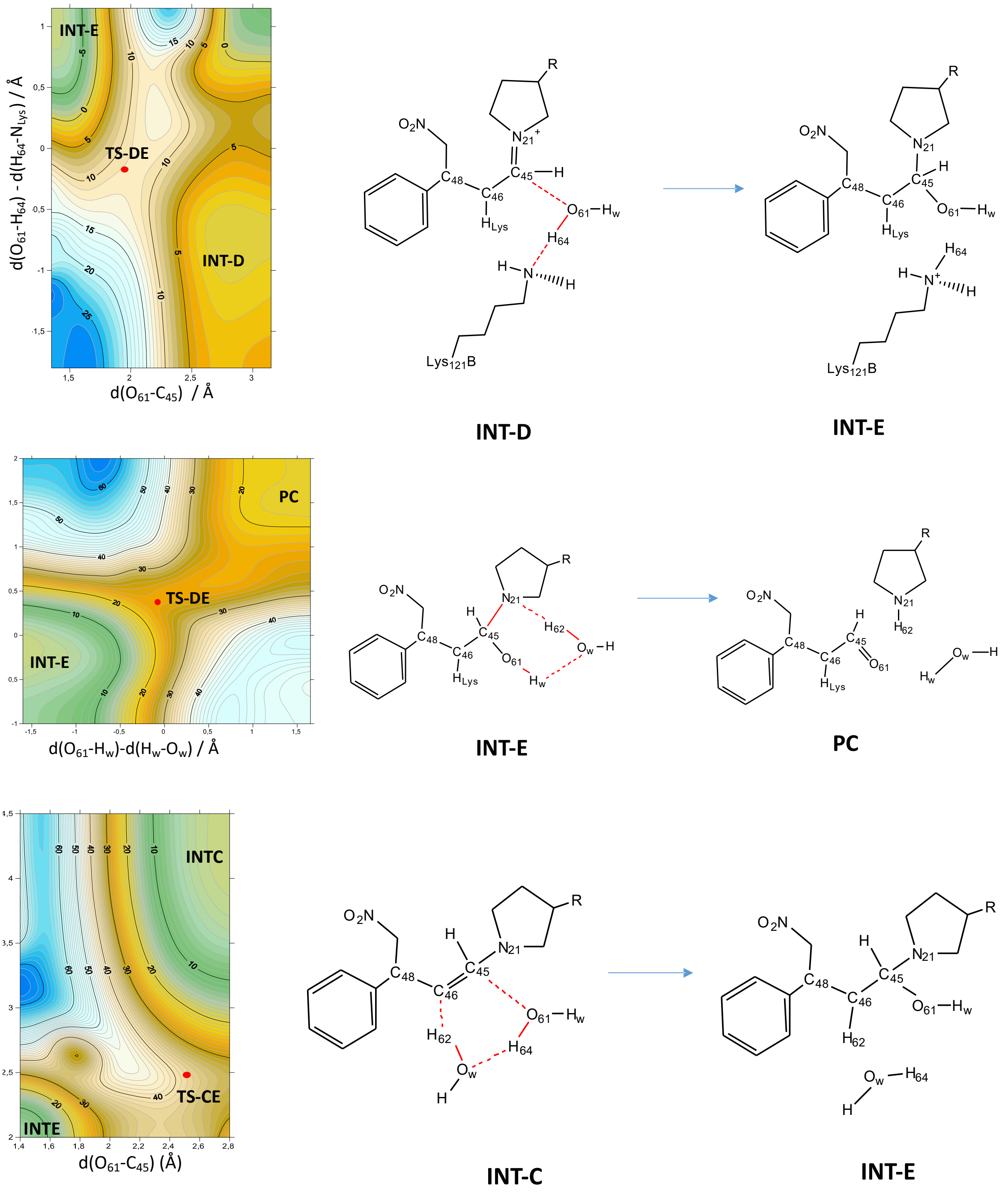

Figure S5c. Free energy surfaces of the INT-D to INT-E, INT-E to PC and INT-C to INT-E chemical steps of the organocatalytic reaction hosted by the T-Sav computed computed in terms of 2D-PMFs at M06-2X:AM1/MM level. Distances are in Å and energies of isoenergetic lines in kcal·mol^{-1}. Position of TSs structures optimized at M06-2X/MM level are indicated as red dots on the surfaces. Schematic representations of every chemical step, indicating in red those bonds that are being formed and/or broken.

Table S7. Averaged key inter-atomic distances (in Å) of every state involved in the reaction derived from the AM1/MM MD simulations of the corresponding windows of the stationary points on the different Free Energy Surfaces.

	C45-N21	O61-Hw	Hw-Ow	Ow-H62	H62-N21	C45-O61	O61-H64	C65-H64	C65-C48	H62-C46	H_{Lys}-C46	N_{Lys}-H_{Lys}	N_{Lys}-H64
**RC**	2.958	2.121	0.969	2.997	1.007	1.242							
**TS-RCA**	1.558	1.072	1.477	1.776	1.069	1.384							
**INT-A**	1.472	0.967	2.531	0.975	4.423	1.445	2.334	1.131	4.146				
**TS-AB**	1.391	0.975		0.973	4.985	1.637	1.100	1.542	4.672				
**INT-B**	1.315	0.967		0.976	3.807	2.811	0.968	3.928	4.252				
**TS-BC**	1.307	0.967		0.974	3.926	2.817	0.969	3.740	2.096				
**INT-C**	1.381	0.969		0.970	3.427	2.809	0.968	4.046	1.540	3.437			
**TS-CE**	1.311	0.975		1.094	2.747	2.510	1.017	3.622	1.489	1.620			
**INT-E**	1.480	0.967	2.585	0.976	3.464	1.425	5.144	3.982	1.532	1.127			
**TS-EPC**	1.521	1.731	1.026	1.164	1.503	1.338	7.008	6.001	1.535				
**PC**	3.030	2.227	0.973	2.322	1.014	1.238	6.927	5.536	1.534				
**INT-C**	1.380	0.968		0.974	5.087	3.686	0.965	3.750	1.534		2.837	1.035	
**TS-CD**	1.332	0.967		0.971	5.341	3.398	0.966	4.400	1.531		1.387	1.376	
**INT-D**	1.304	0.967		0.969	4.780	2.953	0.971	3.573	1.527		1.128	6.340	2.677
**TS-DE**	1.379	0.986		0.976	5.05	1.65	1.083	2.946	1.529		1.128	4.945	1.589
**INT-E**	1.46	0.968		0.976	6.029	1.446	2.188	3.277	1.533		1.125	4.948	1.035

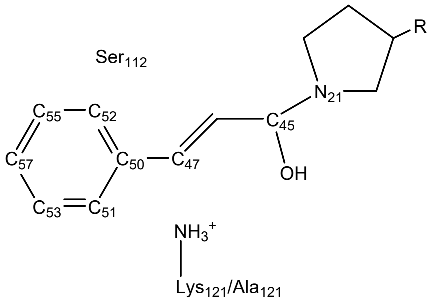

a)

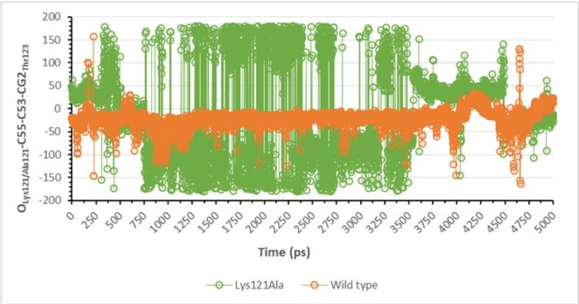

b)

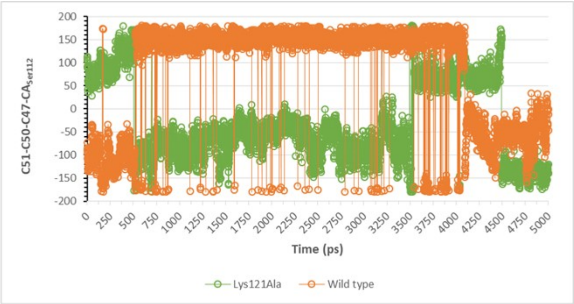

c)

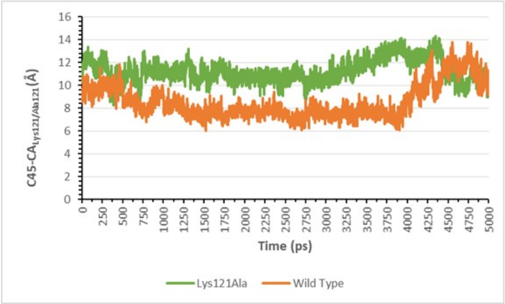

Figure S6. Representation of the substrate displacement along 5 ns of MD simulations for the wild type and the Lys121Ala variant in the INT-A state. **a**) dihedral angle formed between the O atom of Lys121 (wild type) or Ala121 (Lys121Ala mutant), C55-C53 atoms of the ring of the catalyst and CG2 atom of Thr123 in degrees; **b**) dihedral angle formed between the C51-C50-C47 atoms of the ring of the catalyst and CA atom of Ser112 in degrees; and **c**) distance between C45 atom of the ring of the catalyst and CA atom of Lys121 (wild type) or Ala121 (Lys121Ala mutant) in Å

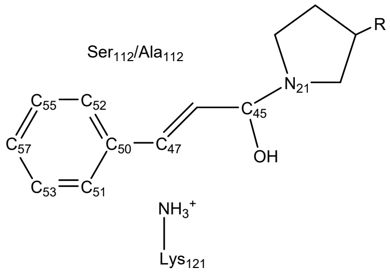

a)

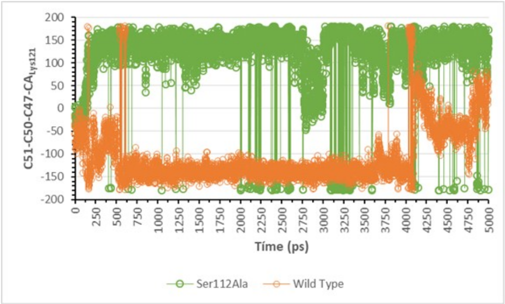

b)

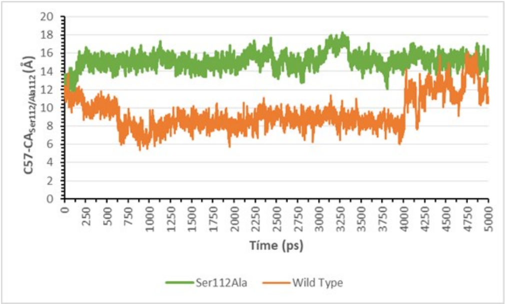

Figure S7. Representation of the substrate displacement along 5 ns of MD simulations for the WT and the Ser112Ala variant in the INT-A state. **a**) dihedral angle formed between C51-C50-C47 atoms of the ring of the catalyst and CA atom of Lys121 in degrees; and **b**) distance between C57 atom of the ring of the catalyst and CA atom of Ser112 (wild type) or Ala112 (Ser112Ala mutant) in Å.

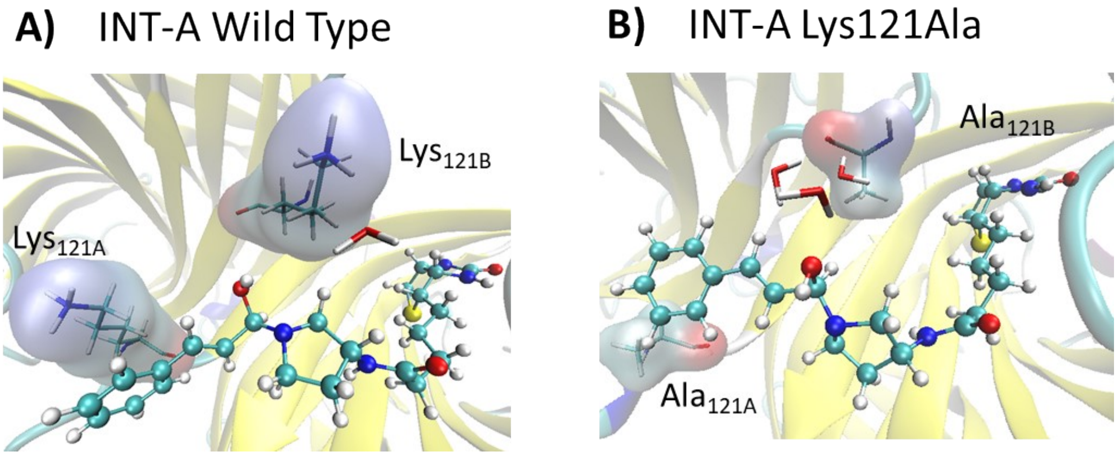

Figure S8. Representation of the active site with special attention to the cavity generated after the Lys121Ala mutation that allow the entrance of additional water molecules on INT-A. **a**) Wild type; and **b**) Lys121Ala mutant.

9 Plasmid maps

9.1 Plasmid map T-rSav (Addgene, Plasmid #17327)

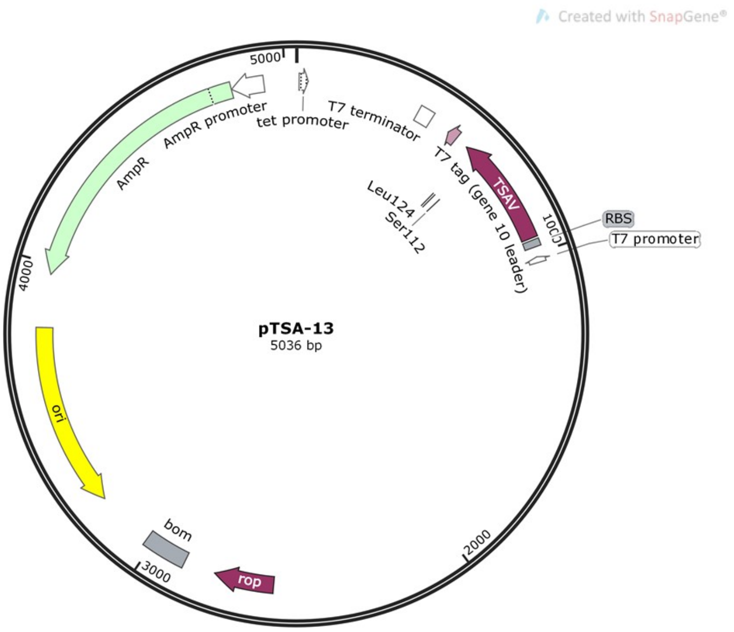

Figure S9. Plasmid used for mutagenesis and recombinant expression of T-rSav.

9.1 Plasmid map M-Sav (Addgene, Plasmid #39860)

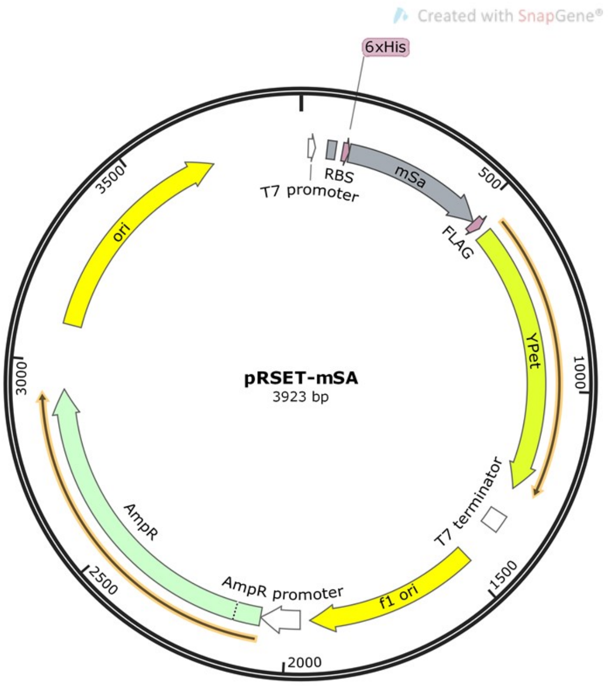

Figure S10. Plasmid used for mutagenesis and recombinant expression of T-rSav.

10 SDS-PAGE Gels of T-rSav and M-Sav

10.1 T-rSav SDS PAGE Gels

T-rSav Wildtype

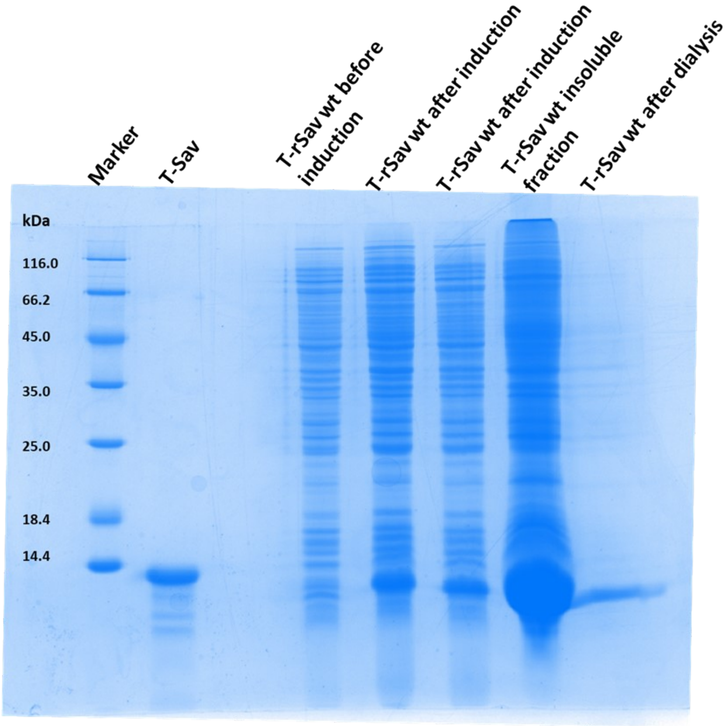

Figure S11. SDS-PAGE gel of T-rSav at different stages of purification and comparison with T-Sav.

T-rSav Exemplary Mutants

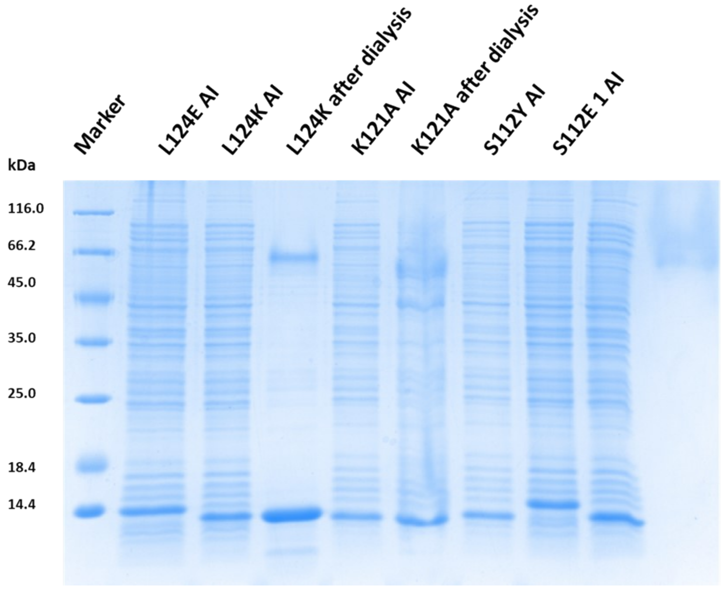

- **Mutant** yield 0.5 to 15 mg/L

Figure S12. SDS-PAGE gel of T-rSav mutants at different stages of purification (AI = after induction).

10.2 M-Sav SDS PAGE Gels

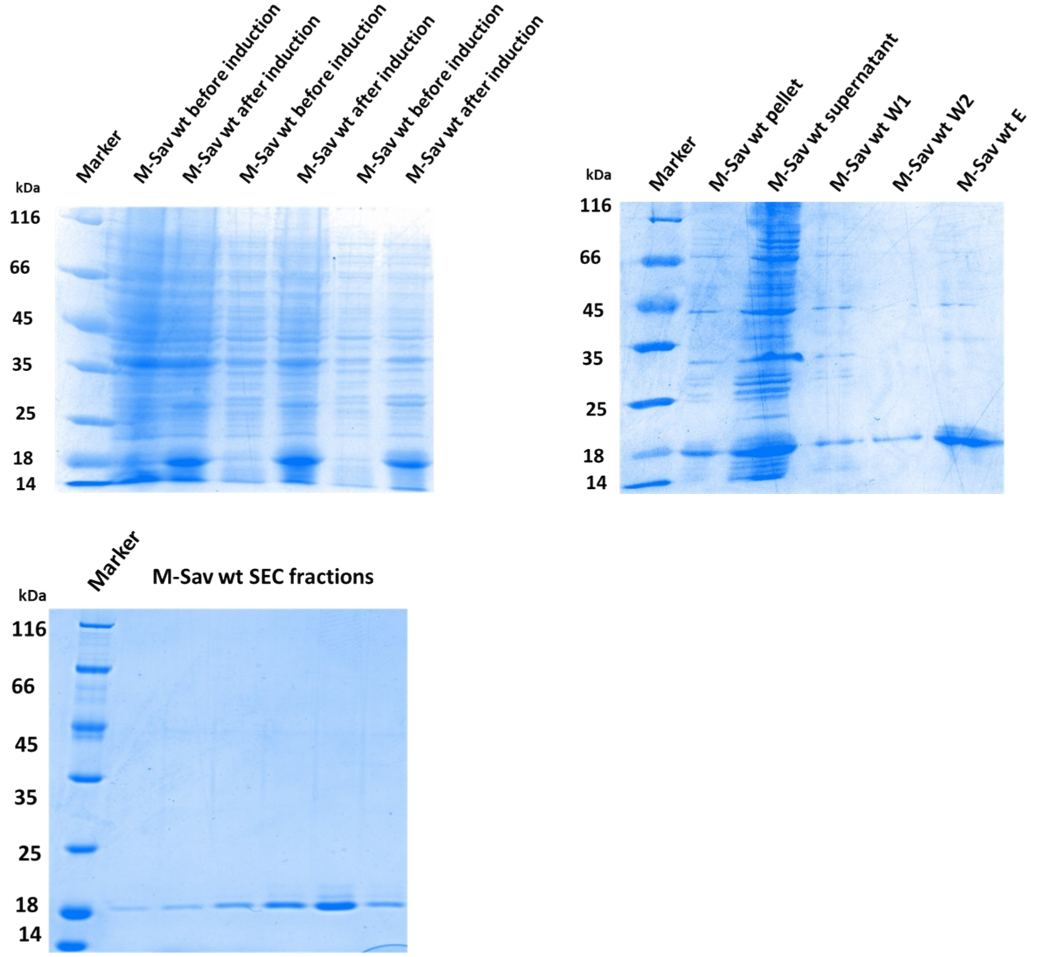

Figure S13. SDS-PAGE gels of M-Sav at different stages of purification.

10.3 D-Sav SDS PAGE Gels

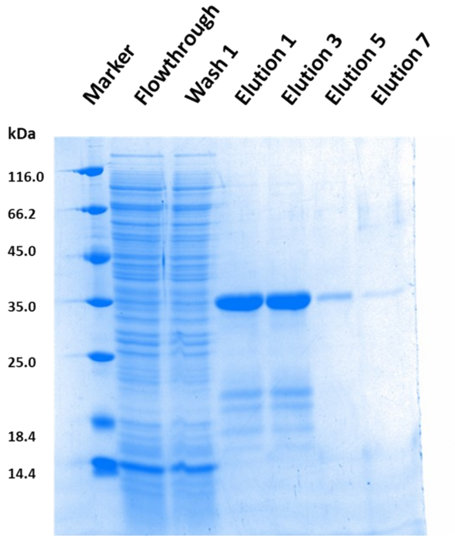

Figure S14. SDS-PAGE gel of D-Sav during Ni-affinity purification.

11 ESI-MS

11.1 ESI-MS of the Expressed T-rSav

Expected MS: 12458.55 Da (M-Met)

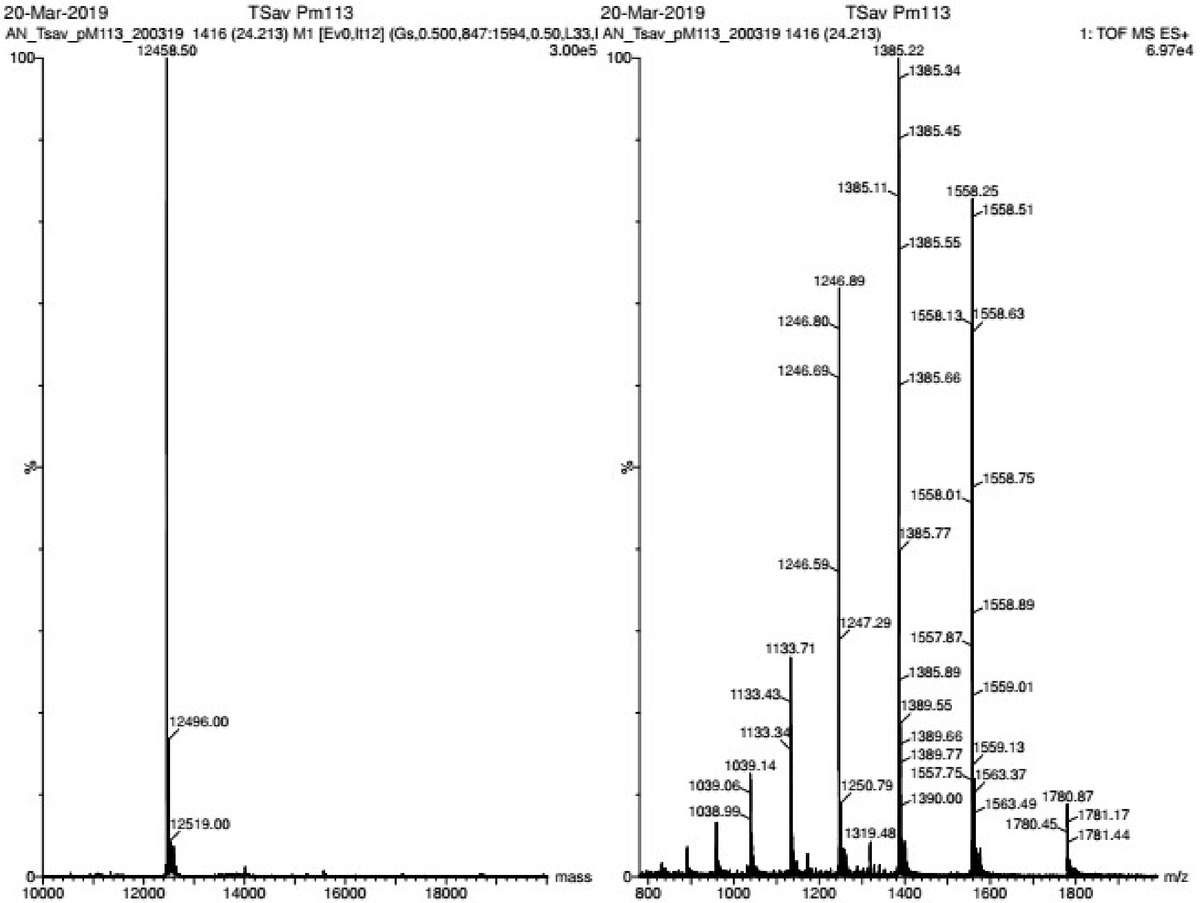

Figure S15. Deconvoluted (left) and raw (right) mass spectrum of T-rSav.

11.2 ESI-MS of the Expressed M-Sav

Expected MS: 15730 Da

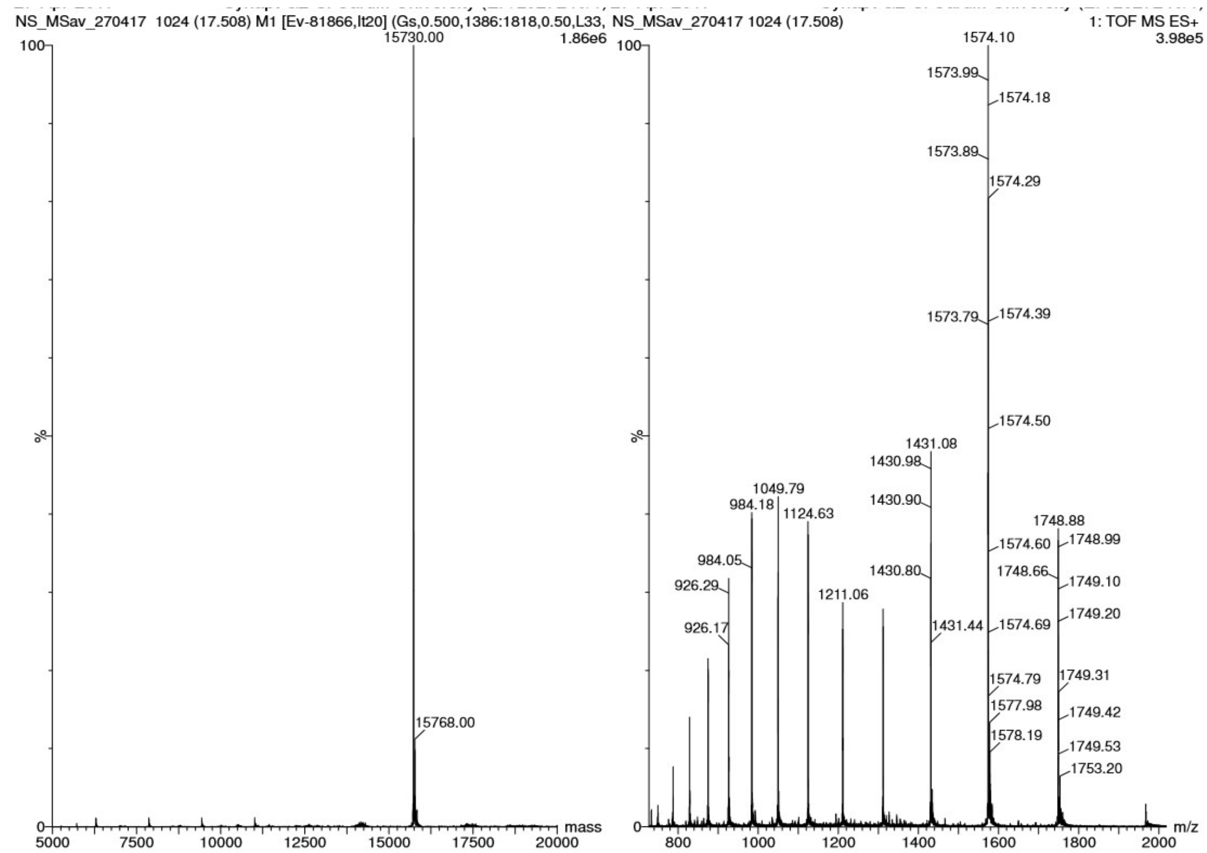

Figure S16. Deconvoluted (left) and raw (right) mass spectrum of M-Sav.

11.3 ESI-MS of the Expressed D-Sav

ESI-MS of D-Savs could not be obtained with the equipment available.[1]

12 CD spectra for M-Sav wt, M-Sav:1, M-Sav:2 and M-Sav:biotin

12.1 CD spectrum M-Sav wt

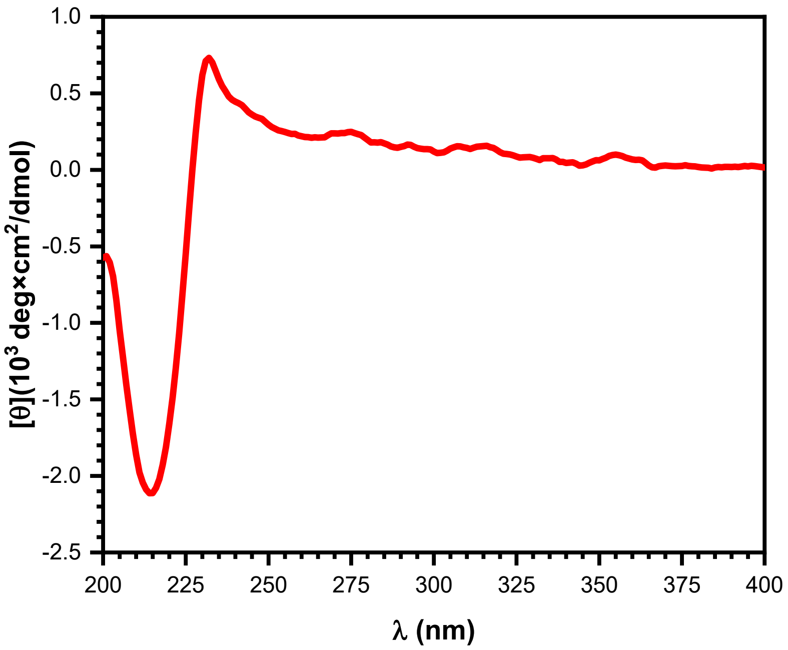

Figure S17. Circular dichroism spectrum of M-Sav wt in PBS, pH 7.4, at 50 µM.

12.2 CD spectrum M-Sav:1

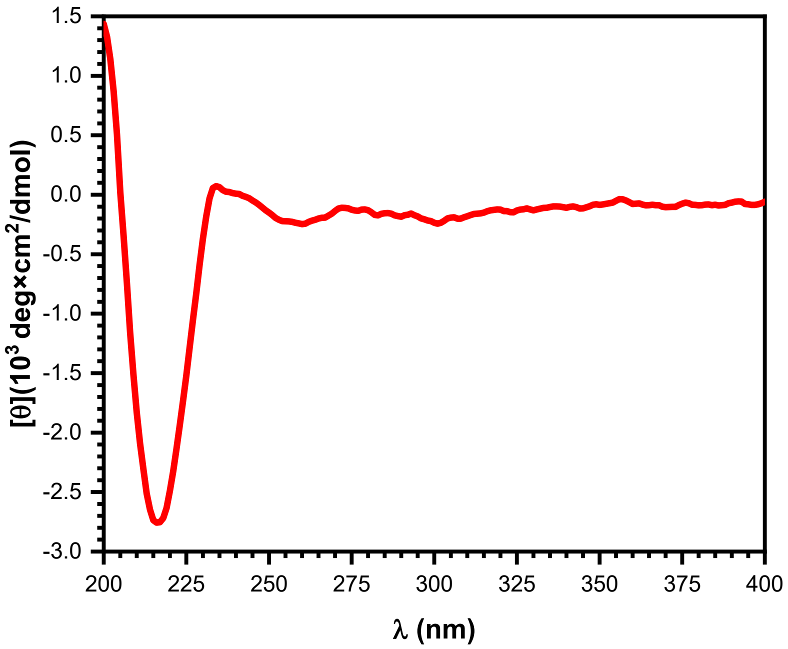

Figure S18. Circular dichroism spectrum of M-Sav wt in PBS, pH 7.4, at 50 µM with 500 µM of **1**.

12.3 CD spectrum M-Sav:2

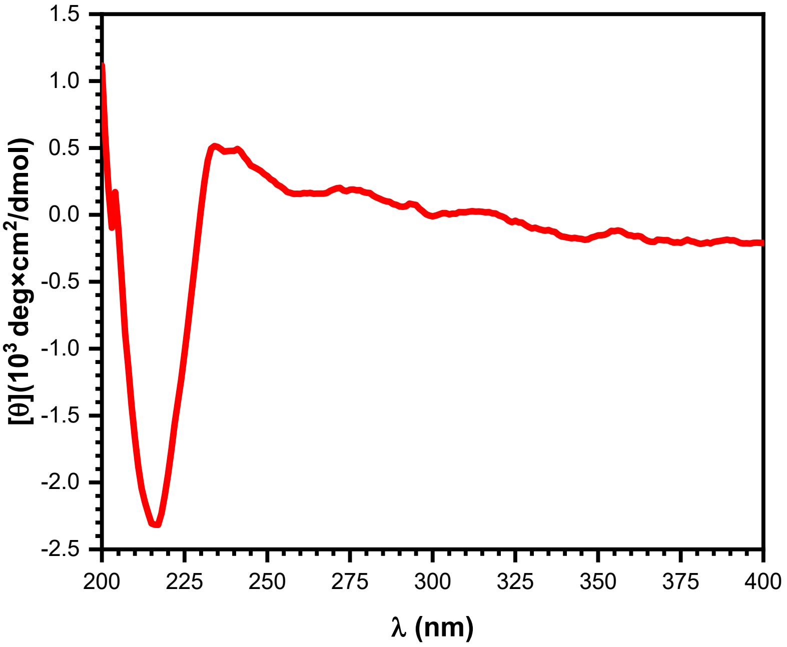

Figure S19. Circular dichroism spectrum of M-Sav wt in PBS, pH 7.4, at 50 µM with 500 µM of **2**.

12.4 CD spectrum M-Sav:biotin

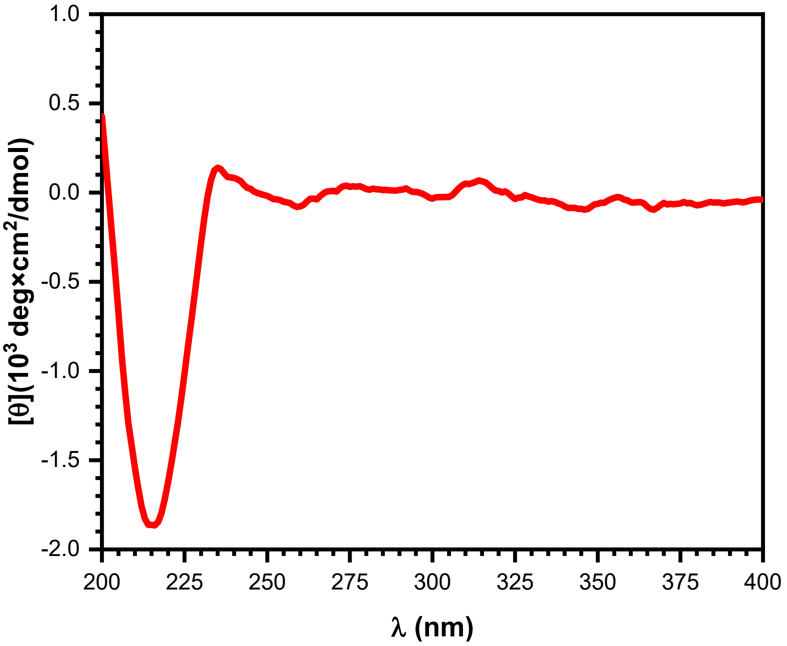

Figure S20. Circular dichroism spectrum of M-Sav wt in PBS, pH 7.4, at 50 µM with 500 µM of **biotin**.

13 Chiral HPLC Data of Activity and Selectivity Screening

Signals in the range from 5 to 10 minutes are impurities from the solvents used for preparative TLC purification. Signals appear higher than usual for S112V and S112Y due to the low amount of product **S2** obtained from catalytic runs. Small shifts in retention time were observed

due to the use of two different machines, column regeneration procedures, and replacement of the guard column. The exact times were confirmed by running the standard **S2** sample before and after each measuring sequence.

13.1 Cinnamaldehyde Runs using Different Streptavidin Constructs

Racemate S2

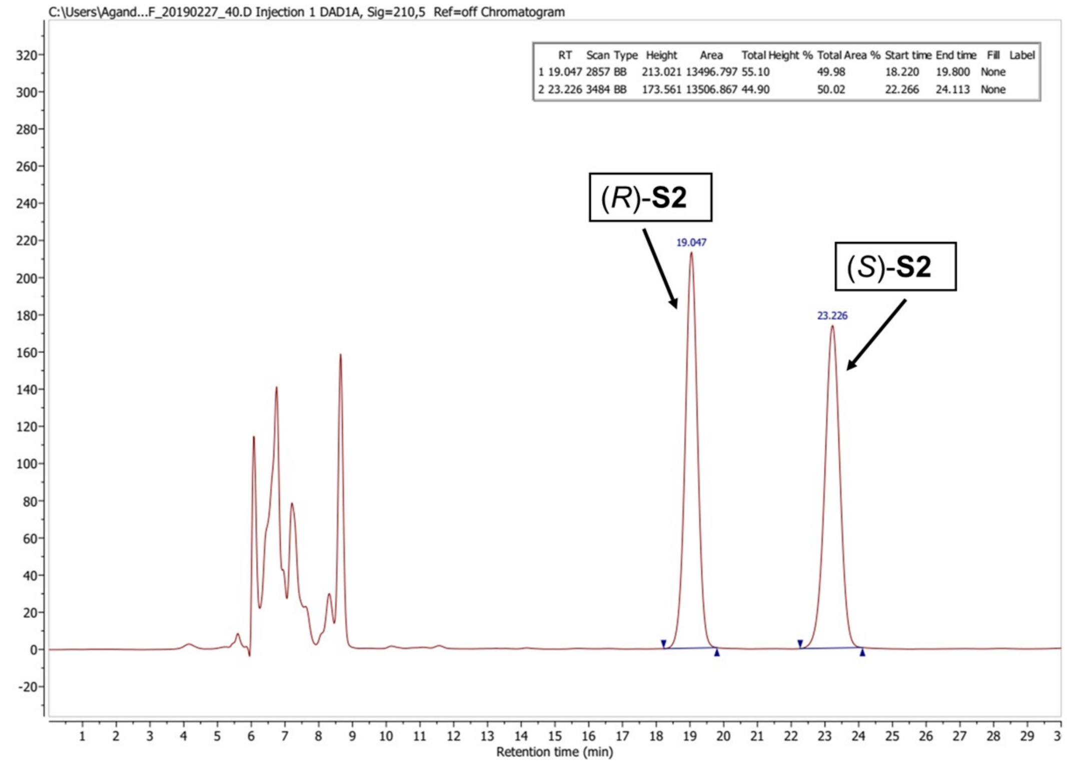

The peaks were assigned based on the known selectivity with the Hayashi-Jørgenson catalyst.[3]

Product S2 with T-Sav:2

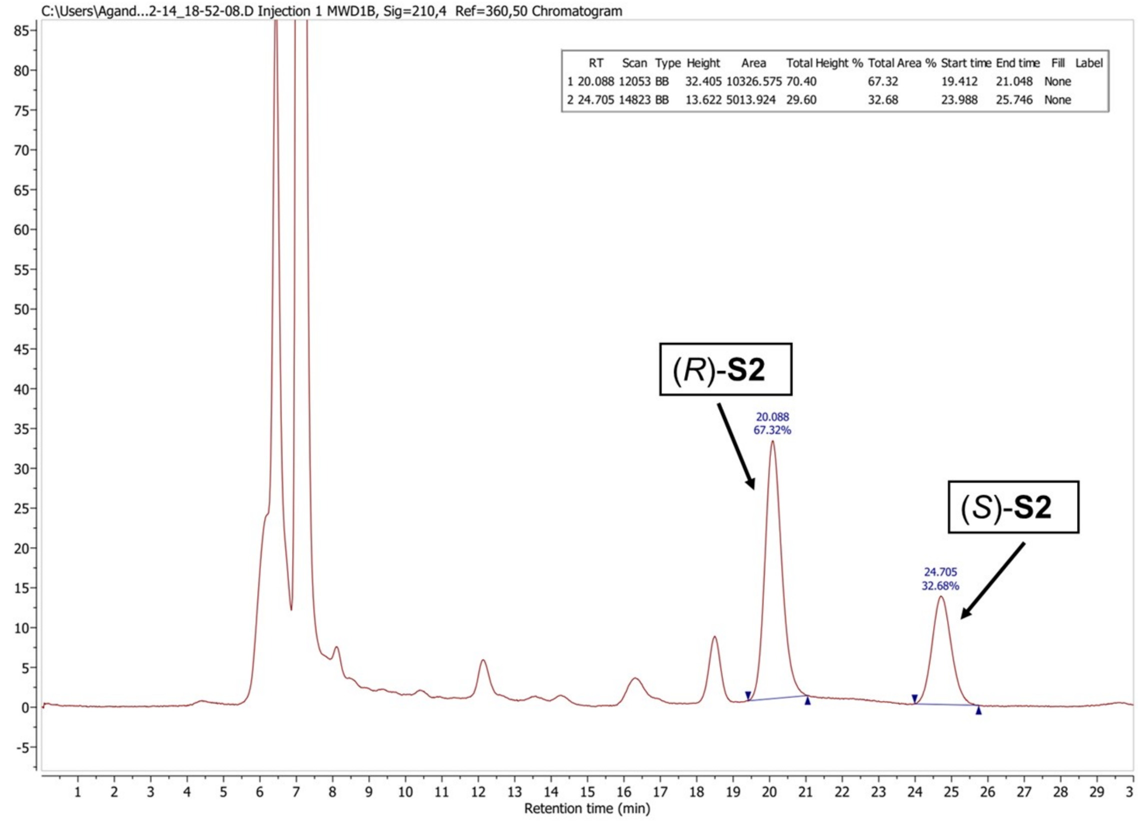

Product S2 with 1

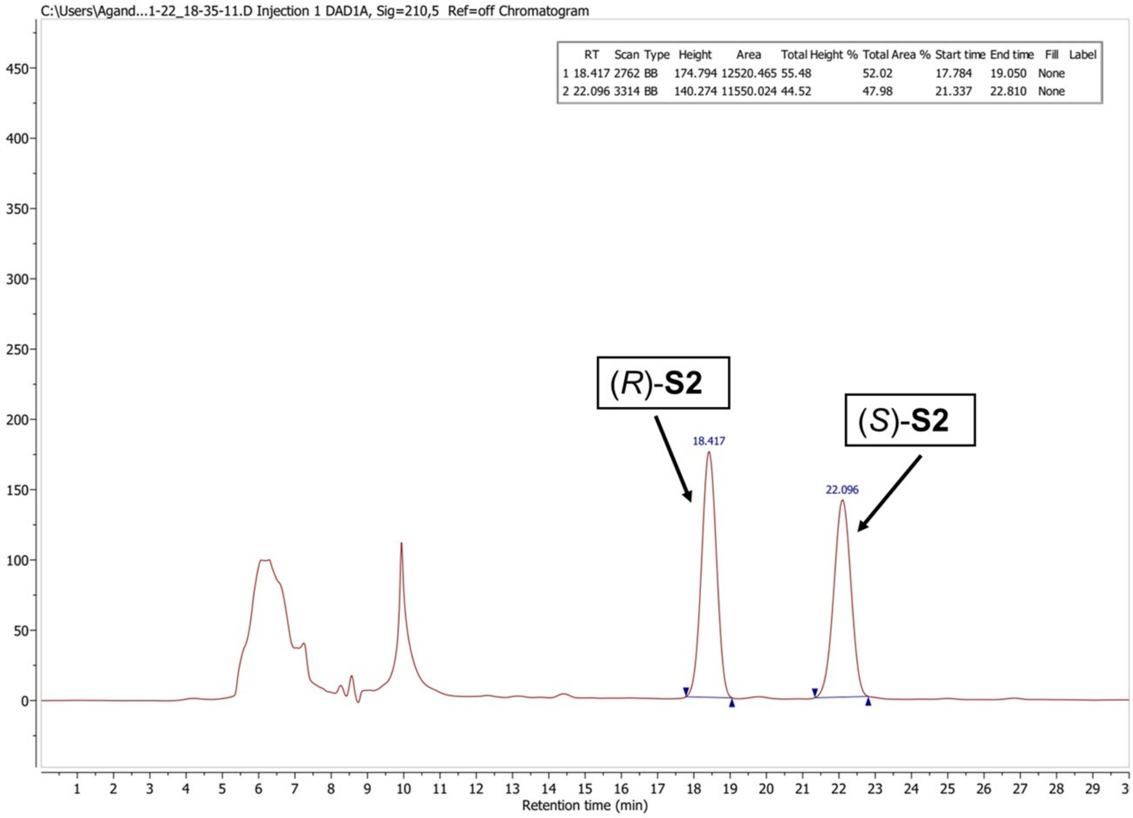

Product S2 with 2

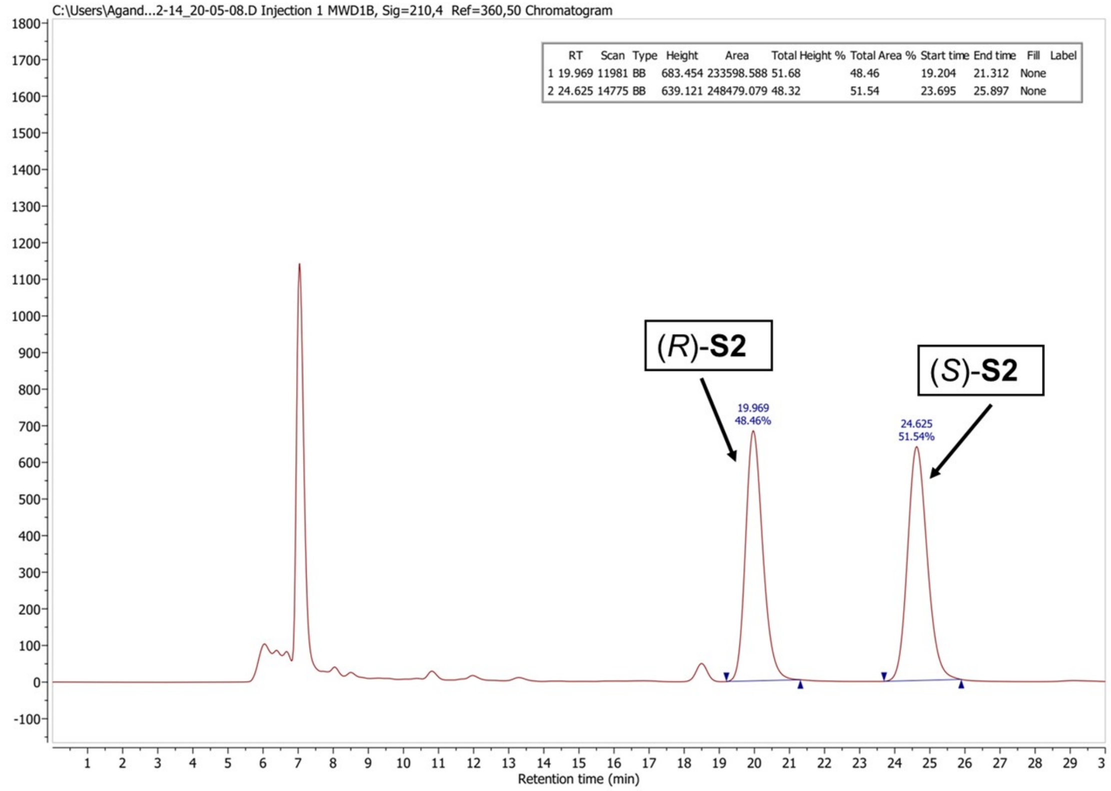

Product S2 with T-rSav:1 (wt)

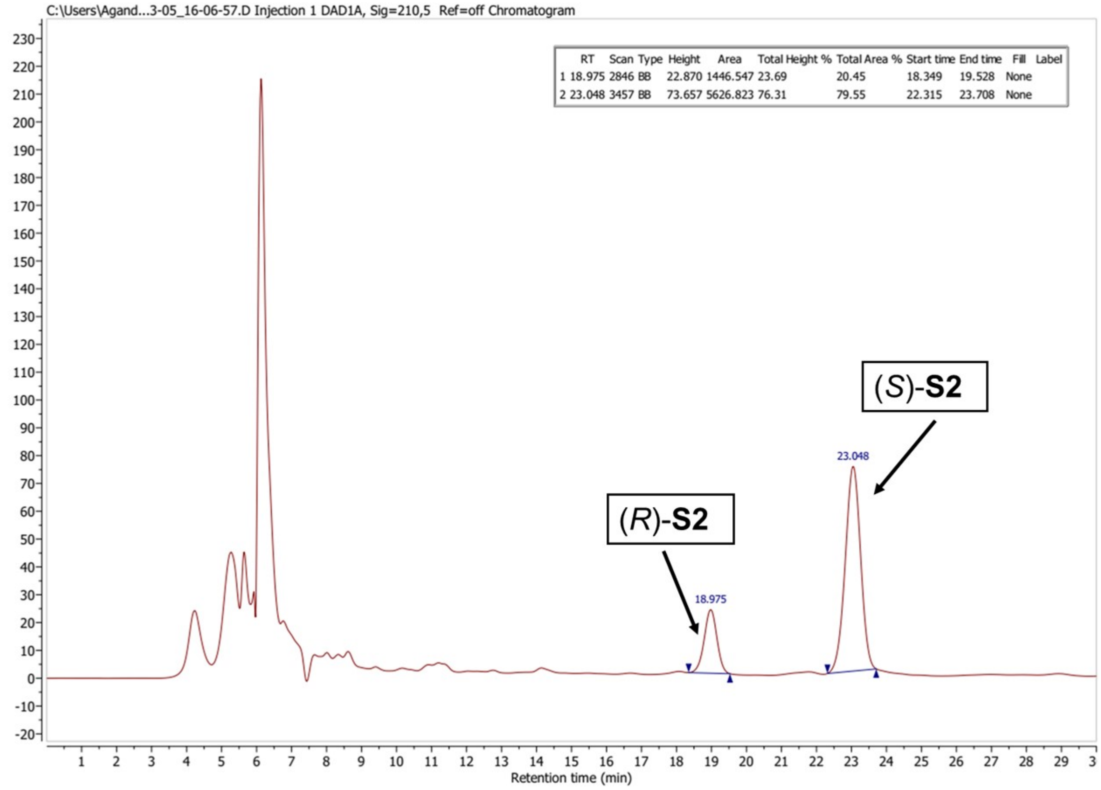

Product S2 with T-rSav:1 (S112V)

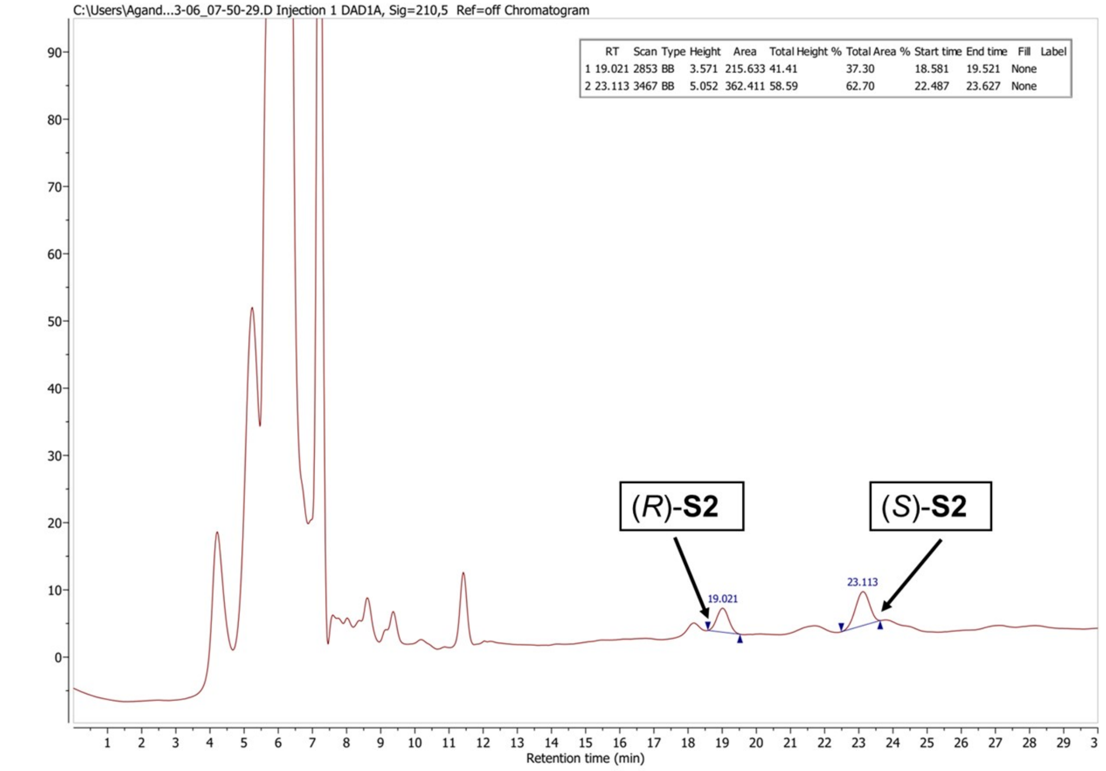

Product S2 with T-rSav:1 (S112E)

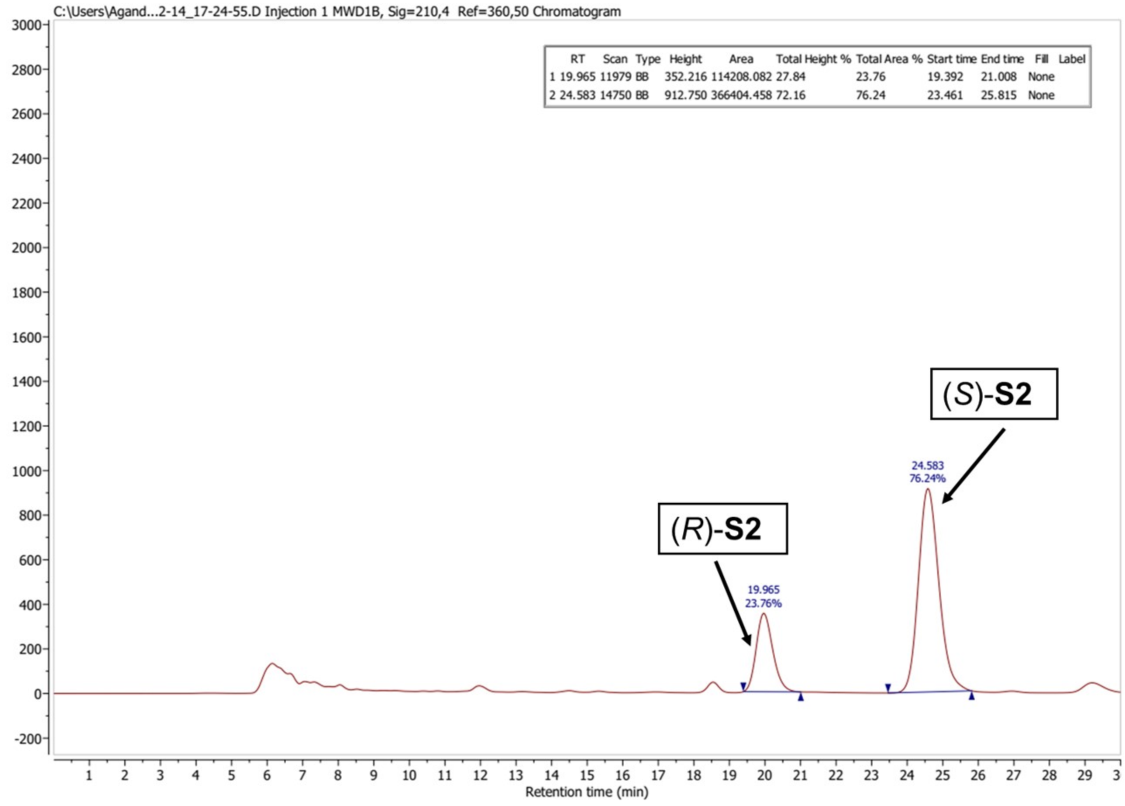

Product S2 with T-rSav:1 (S112Y)

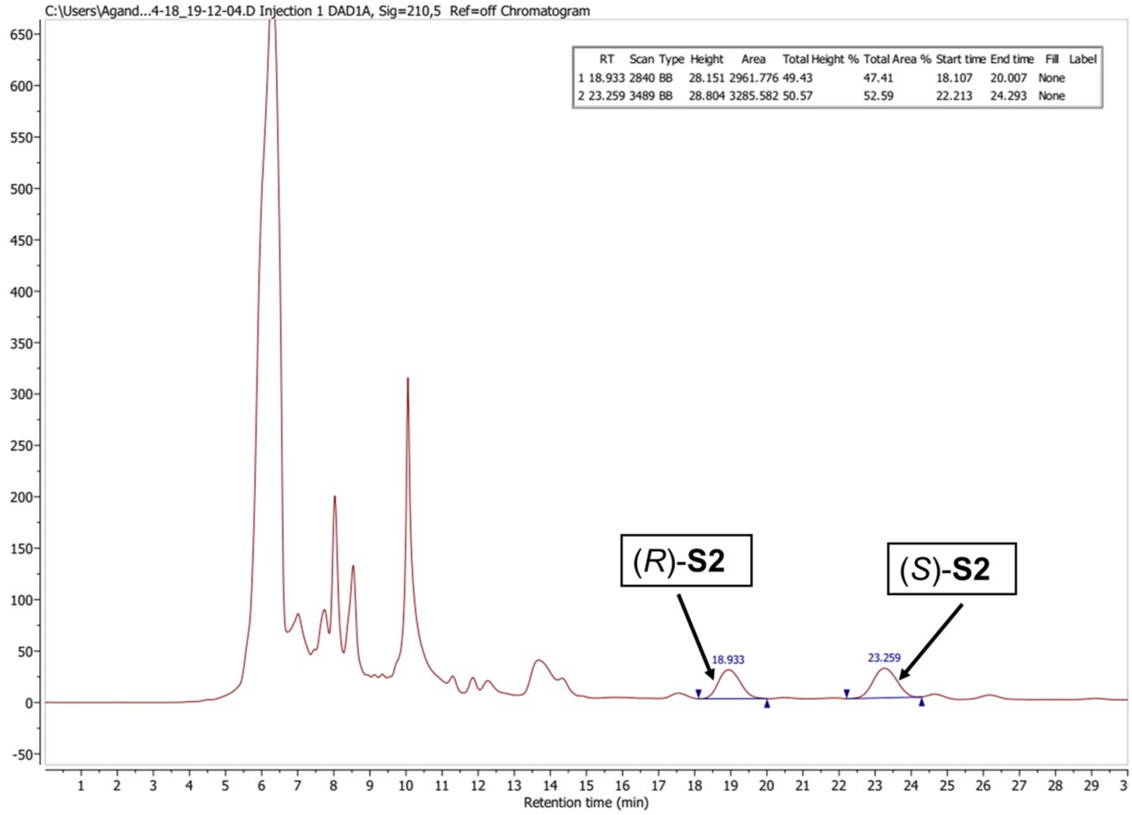

Product S2 with T-rSav:1 (K121A)

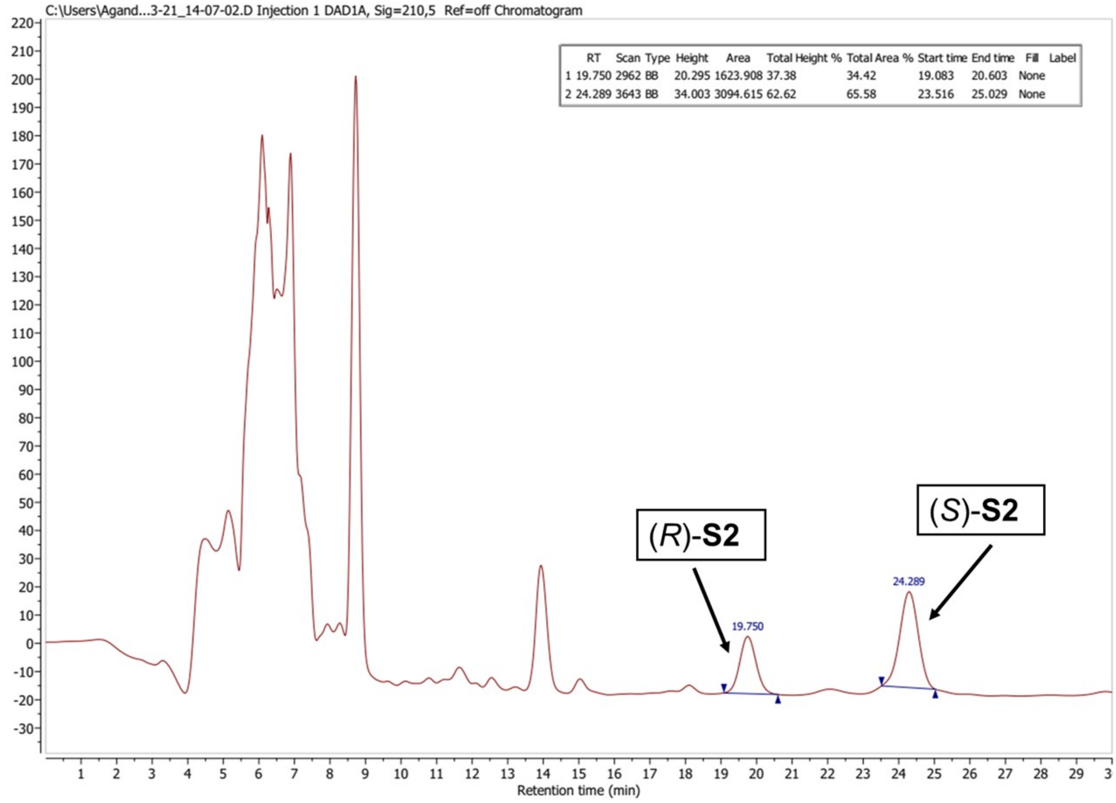

Product S2 with T-rSav:1 (K121M)

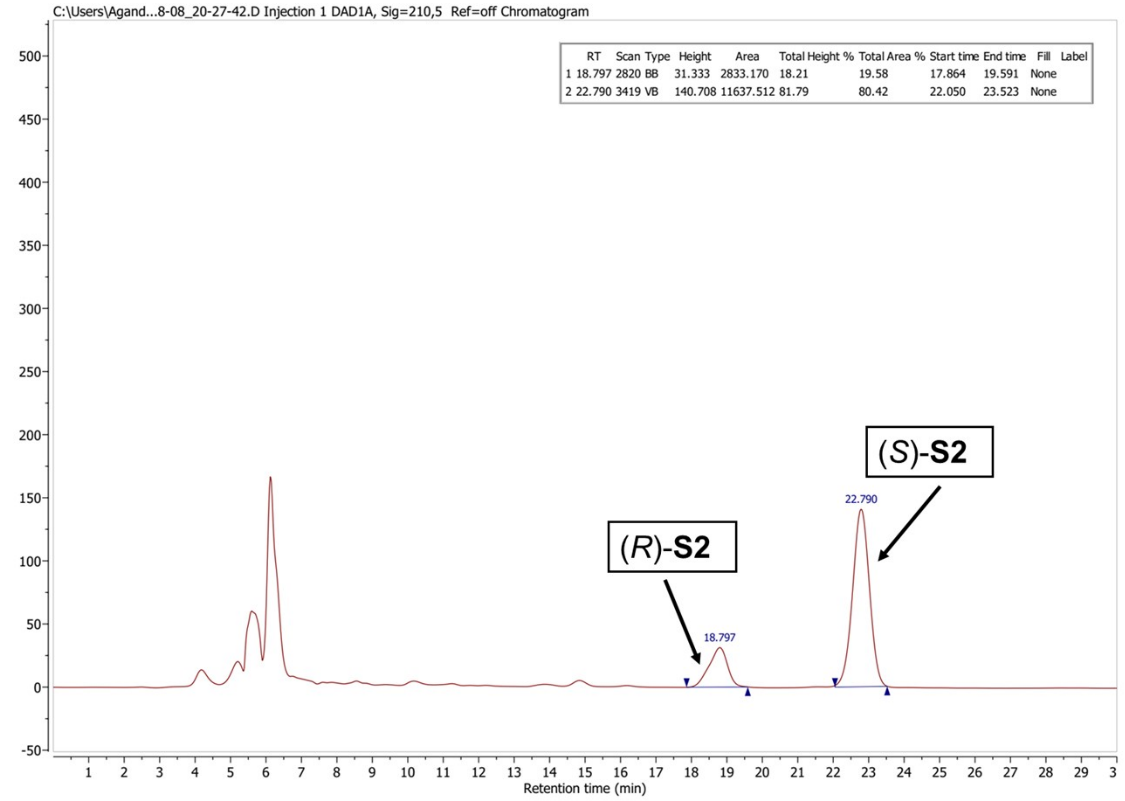

Product S2 with T-rSav:1 (K121R)

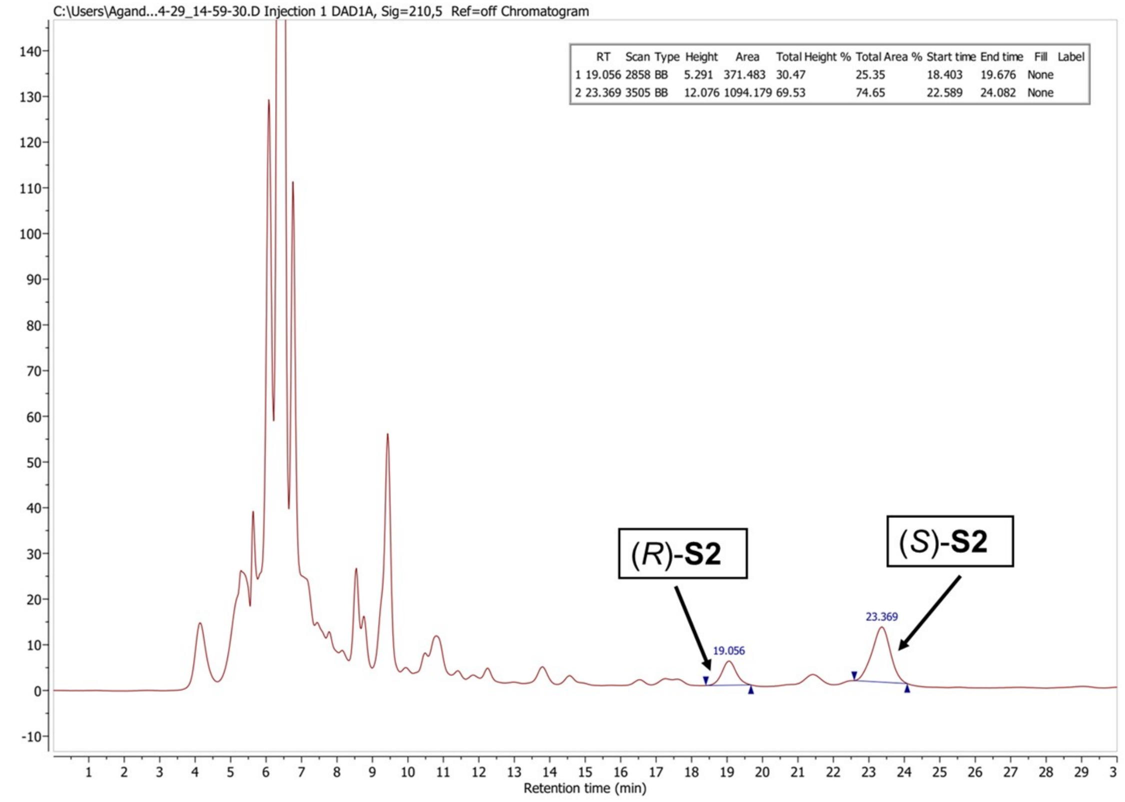

Product S2 with T-rSav:1 (L124E)

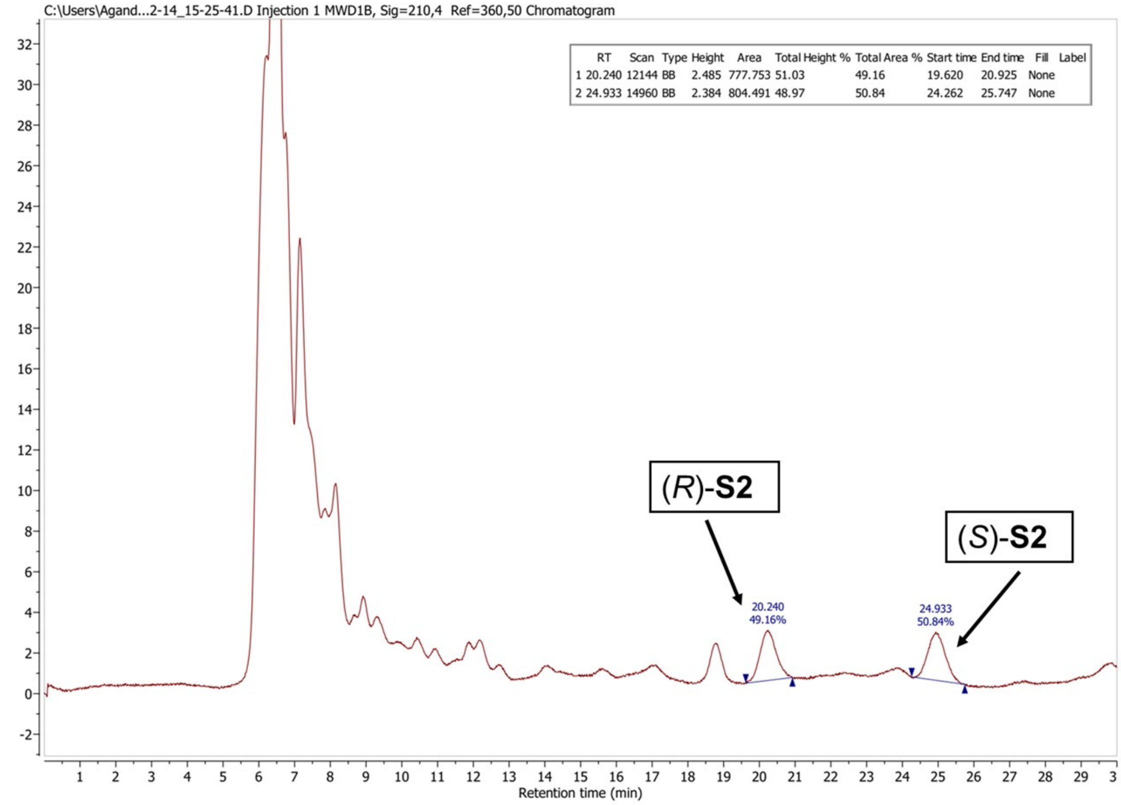

Product S2 with T-rSav:1 (L124K)

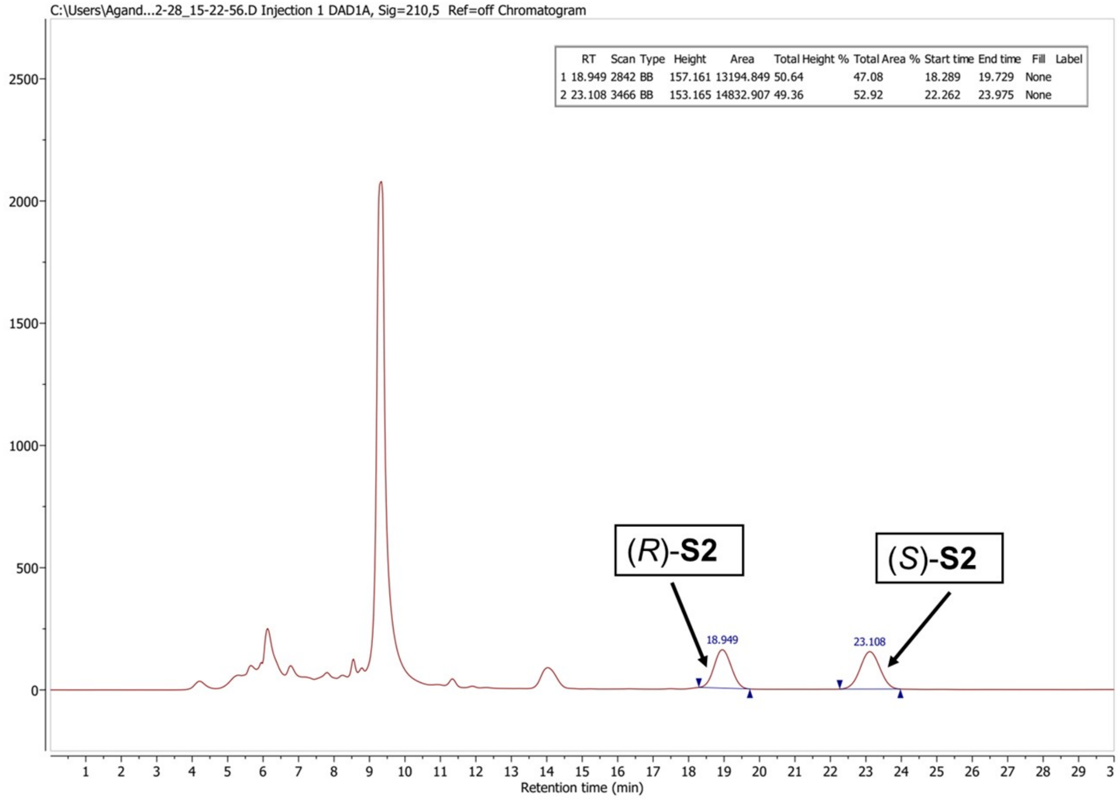

Product S2 with T-rSav:1 (L124W)

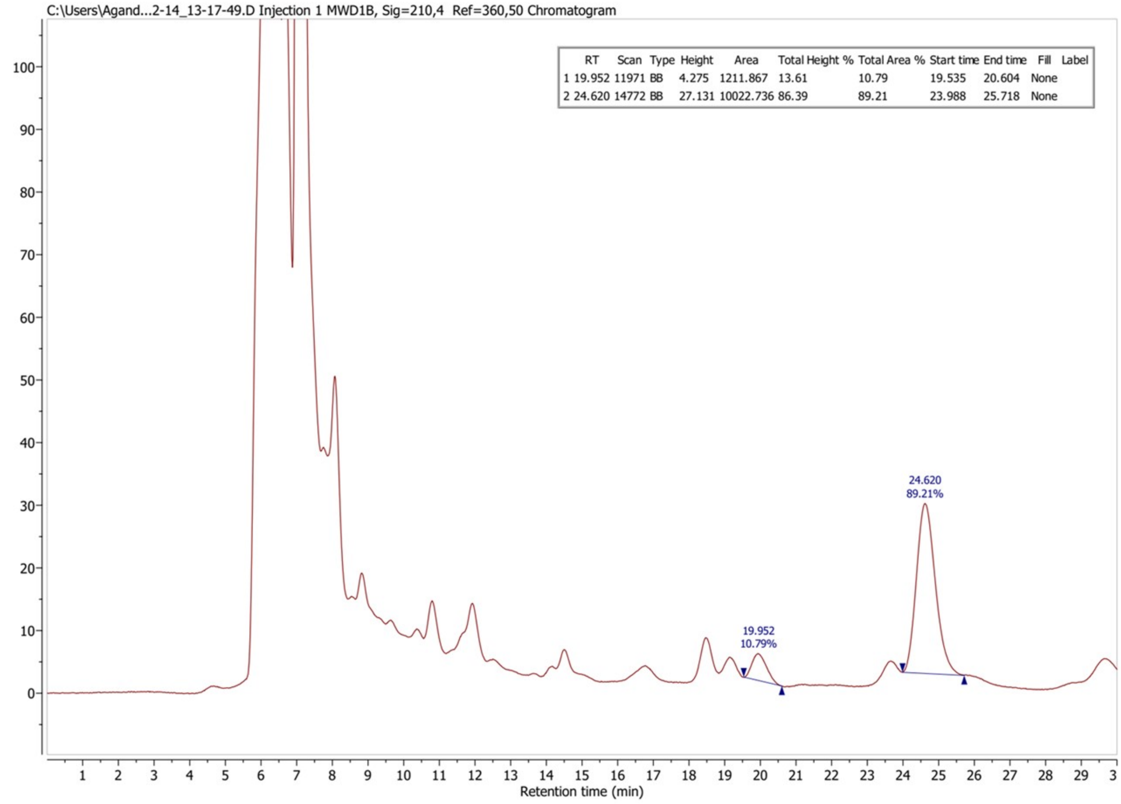

Product S2 with D-Sav:1 (SARK)

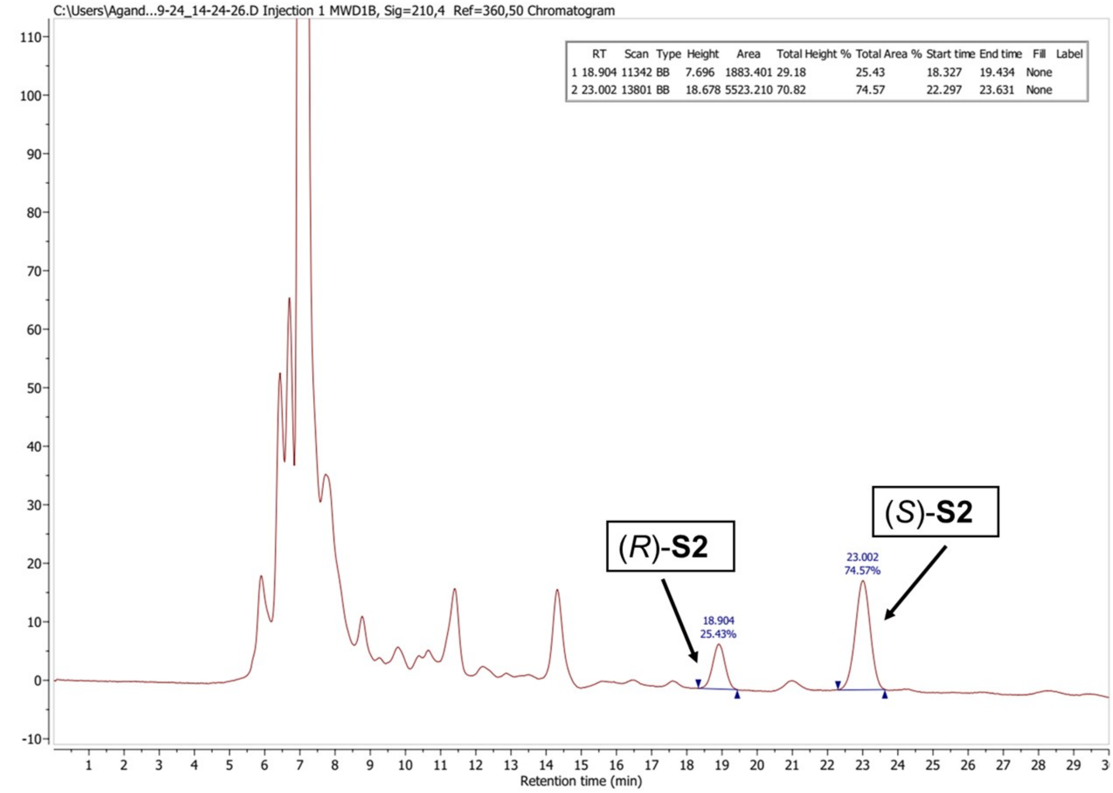

Product S2 with M-Sav:2 (E120L)

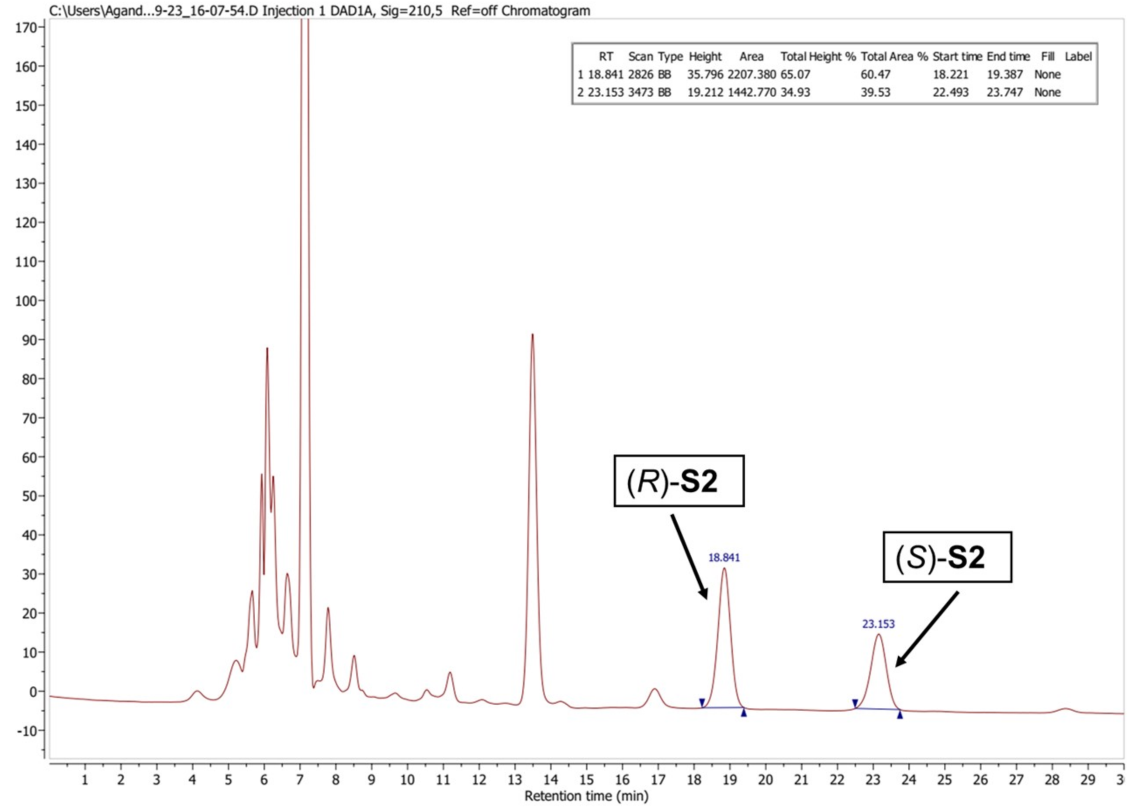

14 Additional Figures & Tables

14.1 Initial Screening Results M-Sav

Table S8. Buffer and pH screening of the model reaction with M-Sav:**1** according to chapter 4.

Buffer	pH	Conversion / % 1,4-addition	Conversion / % 1,2-addition
Tris	7.0	7	12
	7.5	5	21
	8.0	5	56
HEPES	7.0	<1	7
	7.5	2	66
	8.0	2	64
KP_i	7.0	4	7
	7.5	4	39
	8.0	3	53
NaP_i	7.0	3	10
	7.5	<1	58
	8.0	3	63
PBS	7.0	3	3
	7.5	7	13
	8.0	2	46
H_2O	—	2	59

Reactions were run using 1 eq. of cinnamaldehyde (3.3 µmol), 10 eq. of nitromethane (33 µmol), 1 mol% of M-Sav:**1** (33 nmol) in 500 µL of buffer 10 mM at 25 °C for 18 h. Shaking at 300 rpm. Values shown here are mean from triplicate.

Table S9. Buffer strength and co-solvent screening of the model reaction with M-Sav:**1** according to chapter 4.

Buffer	Co-solvent	Conversion / % 1,4-addition	Conversion / % 1,2-addition
KP_i 10 mM	—	4	7
KP_i 25 mM		5	14
KP_i 50 mM		5	10
KP_i 10 mM	EtOAc	3	6
KP_i 10 mM	$CDCl_3$	<1	5
KP_i 10 mM	MeOH	21	13
KP_i 10 mM	MeCN	<1	4

Reactions were run using 1 eq. of cinnamaldehyde (3.3 µmol), 10 eq. of nitromethane (33 µmol), 1 mol% of M-Sav:**1** (33 nmol) in 500 µL of buffer or 250 µL of buffer and 250 µL of co-solvent at 25 °C for 18 h. Shaking at 300 rpm. Values shown here are mean from triplicate.

Table S10. Catalyst and MeOH percentage screening of the model reaction with M-Sav:**1** and M-Sav:**2** according to chapter 4.

Catalyst	MeOH / %	Conversion / % 1,4-addition	Conversion / % 1,2-addition
**M-Sav:1**	0	4	7
	10	9	7
	20	13	9
	30	15	11
	40	18	13
	50	21	13
**M-Sav:2**	0	13	8
	10	16	6
	20	20	7
	30	17	7
	40	19	8
	50	18	13

Reactions were run using 1 eq. of cinnamaldehyde (3.3 µmol), 10 eq. of nitromethane (33 µmol), 1 mol% of M-Sav:**1/2** (33 nmol) in 500 µL KP_i 10 mM/MeOH at 25 °C for 18 h. Shaking at 300 rpm. Values shown here are mean from triplicate.

15 References

1. S. Wu, Y. Zhou, J. G. Rebelein, M. Kuhn, H. Mallin, J. Zhao, N. V. Igareta and T. R. Ward, *J. Am. Chem. Soc.*, 2019, **141**, 15869-15878.
2. G. R. Fulmer, A. J. M. Miller, N. H. Sherden, H. E. Gottlieb, A. Nudelman, B. M. Stoltz, J. E. Bercaw and K. I. Goldberg, *Organometallics*, 2010, **29**, 2176-2179.
3. A. R. Nödling, K. Świderek, R. Castillo, J. W. Hall, A. Angelastro, L. C. Morrill, Y. Jin, Y.-H. Tsai, V. Moliner and L. Y. P. Luk, *Angew. Chem., Int. Ed.*, 2018, **57**, 12478-12482.
4. R. Kowalczyk, P. Kwiatkowski, J. Skarzewski and J. Jurczak, *J. Org. Chem.*, 2009, **74**, 753-756.
5. P. C. Weber, D. H. Ohlendorf, J. J. Wendoloski and F. R. Salemme, *Science*, 1989, **243**, 85-88.
6. M. J. Field, M. Albe, C. Bret, F. Proust-De Martin and A. Thomas, *J. Comput. Chem.*, 2000, **21**, 1088-1100.
7. W. L. Jorgensen, D. S. Maxwell and J. Tirado-Rives, *J. Am. Chem. Soc.*, 1996, **118**, 11225-11236.
8. V. Zoete, M. A. Cuendet, A. Grosdidier and O. Michielin, *J. Comput. Chem.*, 2011, **32**, 2359-2368.
9. J. C. Phillips, R. Braun, W. Wang, J. Gumbart, E. Tajkhorshid, E. Villa, C. Chipot, R. D. Skeel, L. Kalé and K. Schulten, *J. Comput. Chem.*, 2005, **26**, 1781-1802.
10. Y. Duan, C. Wu, S. Chowdhury, M. C. Lee, G. Xiong, W. Zhang, R. Yang, P. Cieplak, R. Luo, T. Lee, J. Caldwell, J. Wang and P. Kollman, *J. Comput. Chem.*, 2003, **24**, 1999-2012.
11. S. E. Feller, Y. Zhang, R. W. Pastor and B. R. Brooks, *J. Chem. Phys.*, 1995, **103**, 4613-4621.
12. M. J. S. Dewar, E. G. Zoebisch, E. F. Healy and J. J. P. Stewart, *J. Am. Chem. Soc.*, 1985, **107**, 3902-3909.
13. J. Baker, *J. Comput. Chem.*, 1986, **7**, 385-395.
14. Y. Zhao and D. G. Truhlar, *Theor. Chem. Acc.*, 2007, **120**, 215-241.
15. M. J. Frisch, G. W. Trucks, H. B. Schlegel, G. E. Scuseria, M. A. Robb, J. R. Cheeseman, G. Scalmani, V. Barone, B. Mennucci, G. A. Petersson, H. Nakatsuji, M. Caricato, X. Li, H. P. Hratchian, A. F. Izmaylov, J. Bloino, G. Zheng, J. L. Sonnenberg, M. Hada, M. Ehara, K. Toyota, R. Fukuda, J. Hasegawa, M. Ishida, T. Nakajima, Y. Honda, O. Kitao, H. Nakai, T. Vreven, J. A. Montgomery, Jr., J. E. Peralta, F. Ogliaro, M. Bearpark, J. J. Heyd, E. Brothers, K. N. Kudin, V. N. Staroverov, R. Kobayashi, J. Normand, K. Raghavachari, A. Rendell, J. C. Burant, S. S. Iyengar, J. Tomasi, M. Cossi, N. Rega, J. M. Millam, M. Klene, J. E. Knox, J. B. Cross, V. Bakken, C. Adamo, J. Jaramillo, R. Gomperts, R. E. Stratmann, O. Yazyev, A. J. Austin, R. Cammi, C. Pomelli, J. W. Ochterski, R. L. Martin, K. Morokuma, V. G. Zakrzewski, G. A. Voth, P. Salvador, J. J. Dannenberg, S. Dapprich, A. D. Daniels, Ö. Farkas, J. B. Foresman, J. V. Ortiz, J. Cioslowski and D. J. Fox, *Journal*, 2009.
16. G. M. Torrie and J. P. Valleau, *J. Comput. Phys.*, 1977, **23**, 187-199.
17. S. Kumar, J. M. Rosenberg, D. Bouzida, R. H. Swendsen and P. A. Kollman, *J. Comput. Chem.*, 1992, **13**, 1011-1021.
